# SOX5 is involved in balanced *MITF* regulation in human melanoma cells

**DOI:** 10.1186/s12920-016-0170-0

**Published:** 2016-02-29

**Authors:** Theresa Kordaß, Claudia E. M. Weber, Marcus Oswald, Volker Ast, Mathias Bernhardt, Daniel Novak, Jochen Utikal, Stefan B. Eichmüller, Rainer König

**Affiliations:** GMP & T Cell Therapy Unit, German Cancer Research Center (DKFZ), INF 280, 69120 Heidelberg, Germany; Integrated Research and Treatment Center, Center for Sepsis Control and Care (CSCC), Jena University Hospital, Erlanger Allee 101, D-07747 Jena, Germany; Network Modeling, Leibniz Institute for Natural Product Research and Infection Biology - Hans Knöll Institute Jena, Beutenbergstrasse 11a, 07745 Jena, Germany; Skin Cancer Unit, German Cancer Research Center (DKFZ), INF 280, 69120 Heidelberg, Germany; Department of Dermatology, Venereology and Allergology, University Medical Center Mannheim, Ruprecht-Karl University of Heidelberg, Mannheim, Germany; Theoretical Bioinformatics, German Cancer Research Center, INF 580, 69121 Heidelberg, Germany

**Keywords:** SOX5, MITF, SOX10, Melanoma, Mixed Integer Linear Programming, Regulatory models

## Abstract

**Background:**

Melanoma is a cancer with rising incidence and new therapeutics are needed. For this, it is necessary to understand the molecular mechanisms of melanoma development and progression. Melanoma differs from other cancers by its ability to produce the pigment melanin via melanogenesis; this biosynthesis is essentially regulated by microphthalmia-associated transcription factor (MITF). MITF regulates various processes such as cell cycling and differentiation. MITF shows an ambivalent role, since high levels inhibit cell proliferation and low levels promote invasion. Hence, well-balanced MITF homeostasis is important for the progression and spread of melanoma. Therefore, it is difficult to use MITF itself for targeted therapy, but elucidating its complex regulation may lead to a promising melanoma-cell specific therapy.

**Method:**

We systematically analyzed the regulation of MITF with a novel established transcription factor based gene regulatory network model. Starting from comparative transcriptomics analysis using data from cells originating from nine different tumors and a melanoma cell dataset, we predicted the transcriptional regulators of MITF employing ChIP binding information from a comprehensive set of databases. The most striking regulators were experimentally validated by functional assays and an *MITF-*promoter reporter assay. Finally, we analyzed the impact of the expression of the identified regulators on clinically relevant parameters of melanoma, i.e. the thickness of primary tumors and patient overall survival.

**Results:**

Our model predictions identified SOX10 and SOX5 as regulators of *MITF*. We experimentally confirmed the role of the already well-known regulator SOX10. Additionally, we found that SOX5 knockdown led to *MITF* up-regulation in melanoma cells, while double knockdown with SOX10 showed a rescue effect; both effects were validated by reporter assays. Regarding clinical samples, *SOX5* expression was distinctively up-regulated in metastatic compared to primary melanoma. In contrast, survival analysis of melanoma patients with predominantly metastatic disease revealed that low *SOX5* levels were associated with a poor prognosis.

**Conclusion:**

*MITF* regulation by SOX5 has been shown only in murine cells, but not yet in human melanoma cells. SOX5 has a strong inhibitory effect on MITF expression and seems to have a decisive clinical impact on melanoma during tumor progression.

**Electronic supplementary material:**

The online version of this article (doi:10.1186/s12920-016-0170-0) contains supplementary material, which is available to authorized users.

## Background

Normal melanocytes located in the *stratum basale* of the epidermis are beneficial cells that are capable of producing the pigment melanin; these cells transfer melanin to keratinocytes and by this means prevent DNA damage which can be caused by ultraviolet radiation. However, melanocytes can transform into malignant cells. Melanoma cells exhibit an imbalanced regulation that allows for abnormally high proliferation rates, reduced apoptosis and the potential to form metastases. Melanoma is the most lethal form of skin cancer and causes approximately 75 % of all skin cancer deaths, with a rising incidence rate in the last three decades [1–3]. Although the resection of early diagnosed melanoma yields very high curation rates, for progressed melanoma, no effective therapy is currently available. Common tumor treatments like radiotherapy and chemotherapy often fail for the treatment of patients with metastatic melanoma, and the average survival rate for these patients is less than 1 year [3, 4]. To improve treatment for therapy, it is mandatory to better understand the molecular pathways and transcriptional regulation involved in melanoma formation. In particular, changes in the transcriptional regulation driving melanoma progression and metastasis are crucial to find new strategies to cure melanoma patients [5, 6].

We focused our work on the so-called master regulator of melanocytes and melanoma cells, microphthalmia-associated transcription factor (MITF) [7]. MITF is a basic-helix-loop helix leucine zipper transcription factor that binds as a dimer to conserved sequences of the E-box (CATGTG) and M-box (AGTCATGTGCT) motifs in the promoter region of its target genes. MITF regulates several genes involved in melanocyte differentiation, proliferation and it also regulates the expression of the two pacemaker enzymes of melanogenesis, tyrosinase (TYR) and dopachrome tautomerase (DCT) [8, 9]. Most melanoma cancer cells maintain their ability to produce melanin, and often genes of melanogenesis are highly expressed. These characteristics distinguish melanoma cells from other cancer cells and melanogenesis is a discussed target for chemotherapy [10].

Different *MITF* expression levels have been shown to result in very divergent clinical courses in melanoma patients. Low *MITF* expression levels can be observed in invasive melanoma and are therefore associated with a low survival rate [11]. On the contrary, high *MITF* expression levels can slow down the proliferation of melanoma cells [12]. Cancer cells are characterized by an abnormally high proliferation rate and they circumvent cell cycle stagnancy and apoptosis. A strategy of melanoma cells to gain a high proliferation rate is to avoid high *MITF* expression levels, which have an anti-proliferative effect. Besides this, many melanoma tumors (~50 %) exhibit a driving mutation in the serine/threonine-protein kinase B-RRAF (BRAF) [13, 14]. The mutation results in a constantly activated kinase that permanently stimulates extracellular-signal regulated protein kinase 2 (ERK2), which in turn phosphorylates and targets MITF proteins for ubiquitin-dependent degradation via the proteasomal pathway [15] and thereby decreases the activity of MITF. Hoek and coworkers found that MITF levels can be used as a marker to distinguish proliferative and invasive phenotypes of melanoma cell lines with low MITF levels marking the invasive state [12, 16, 17].

The aim of this study was to further investigate the regulation of *MITF* and the impact of *MITF* regulators on melanoma progression. The transcriptional regulation of *MITF* is very complex, involving numerous activating and inhibiting factors. For example, SRY (sex determining region Y)-box 10 (SOX10), paired box 3 (PAX3) [18] and one cut homeobox 2 (ONECUT2) [19] activate *MITF* expression, whereas zinc finger E-box-binding homeobox 1 (ZEB1) [20] and GLI family zinc finger 2 (GLI2) [21] repress *MITF* expression.

We applied a computational approach we developed earlier [22] to identify *MITF* transcriptional regulators that could predict changes in *MITF* expression levels over a set of different cancer types (NCI-60 panel) and a set of melanoma samples. The next step was to verify the effect of the obtained regulators on *MITF* mRNA levels using siRNA transfection and an *MITF*-promoter reporter assay. Finally, we unraveled the relationships between the obtained transcription factor expression levels and central clinical parameters like overall survival and the Breslow thickness.

## Methods

### Gene regulatory network models to identify regulators of *MITF*

We developed a transcription factor (TF) *MITF* target gene regulatory network model. We wanted to identify TFs that best explain different *MITF* expression levels over a set of different cell lines. For this, we predicted the expression of the *MITF* gene in a sample *j* by minimizing the differences *e*_mitf,j_ between real *MITF* expression *g*_mitf,j_ and predicted expression ${\tilde{g}}_{\mathrm{mitf},\mathrm{j}}$ realized by the constraints

1 $$ {g}_{\mathrm{mitf},\mathrm{j}}-{\tilde{g}}_{\mathrm{mitf},\mathrm{j}}-{e}_{\mathrm{mitf},\mathrm{j}}\le 0 $$

2 $$ -{g}_{\mathrm{mitf},\mathrm{j}}+{\tilde{g}}_{\mathrm{mitf},\mathrm{j}}-{e}_{\mathrm{mitf},\mathrm{j}}\le 0 $$

The predicted *MITF* expression was based on the linear equation

3 $$ {\tilde{g}}_{\mathrm{i},\mathrm{j}}={\beta}_0+{\displaystyle {\sum}_{\mathrm{t}=1}^T{\beta}_t\cdot {es}_{t,i}\cdot {eff}_{t,j}} $$

with an additive offset β_0_. T is the number of all TFs with available information on target genes, β_t_ is the optimization parameter for TF t, and es_t,i_ was calculated from an integration of binding data in order to only account for TFs that are known or predicted to regulate *MITF.* es_t,i_ is equal to or greater than 1 if we had experimental and computational evidence of TF t binding to gene i using the databases Metacore™ (http://thomsonreuters.com/metacore/), ChEA [23], Encode (http://www.genome.gov/Encode/) and Transcription Factor Binding Affinity (TBA) [24] (details are given in the next section and at [22]). *eff*_*t,j*_ is the effect of TF t in sample j and was calculated by the activity of a TF based on its cumulative effect on its target genes, i.e.

4 $$ {eff}_{tj}={act}_{tj}=\frac{{\displaystyle {\sum}_{\mathrm{i}=1}^n}{es}_{ti}\cdot {g}_{\mathrm{i}\mathrm{j}}}{{\displaystyle {\sum}_{\mathrm{i}=1}^n}{es}_{ti}} $$

With the use of a branch and cut based optimization program (Gurobi™ 5.5, http://www.gurobi.com/) to solve the Mixed Integer Linear Programming (MILP) problem, the β-coefficients were calculated in order to minimize the sum of differences between measured and predicted *MITF* expression for all samples (objective function). The *MITF* model was restricted to a defined number of regulators from the set of all putative regulators (19 TFs). We applied a bottom-up approach to identify the most important regulators of the model, starting with restricting the model to one regulator. Within each of the following runs, one additional regulator was added to the model. The optimizer selected independently in every run the best regulators in order to minimize the objective function. The prediction performance of each model was estimated by the correlation between real and predicted *MITF* expression in the test data (unseen data, not used for learning the model) based on a leave-one-out cross validation (LOO-CV). For details regarding the MILP model and activity definition see Schacht et al. [22].

### Binding evidence

As described previously [22], we used several sources to assess TF binding information. From the database MetaCoreTM (http://thomsonreuters.com/metacore/) human TF-target gene interactions were selected, of both of the categories direct and indirect. Additionally, we used z-scores of the Total Binding Affinity (TBA) which are calculated TF binding profiles for the whole promoter based on position weight matrices [24, 25]. Moreover, human entries of the CHIP Enrichment Analysis (ChEA) database were used containing large data sets of high-throughput chromatin immunoprecipitation experiments [23]. At the date of analysis (July 2013) the ChEA database for man comprised of 83 transcription factors, 20,035 genes and 131,996 total entries. In addition, we used chromatin immunoprecipitation data from the ENCODE project (http://www.genome.gov/Encode/). We used binding information of cell lines for which the most comprehensive set of regulation information was available (Tier 1). Binding of a transcription factor to a target gene as listed in Encode, was scored as “1” or if absent, as “0”, respectively. Target genes occurring more than once, were combined in single rows containing consistent (intersecting) hits and transcription factors showing up multiple times were assembled into one column as the union of hits. Information on regulatory transcription factor/target gene interaction was considered reliable if (i) this pair was found in Metacore with the annotation “direct”, or if (ii) this pair was found in at least two of the datasets Metacore “indirect”, CheA, Encode and TBA with a value greater or equal to one. For these TF/target gene pairs, their putative regulatory interaction was denoted edge strength es_t,i_ between TF t and target gene i, and set to the number of occurrences of the specific TF/target gene combinations among the datasets CheA, Metacore “direct” activation, Metacore “direct inhibition”, Metacore “indirect activation” and Metacore “indirect inhibition”. TBA values greater or equal to one were added to the edge strength. For all TF/target gene pairs missing criteria (i) or (ii), the edge strength was set to zero, i.e. this TF/target gene pair was not considered by our prediction algorithms. The binding information of SOX5/MITF interaction was taken from Metacore where it was annotated as “direct inhibition”. In addition, the z-score of TBA of SOX5 binding to the human MITF promoter was strongly positive (z = 1.5, see Additional file 1: Figure S1).

### Gene expression data

To identify prominent transcription factors of *MITF* with our regulatory network model, we used the gene expression profiles of 59 cancer cell lines from the National Cancer Institute (NCI-60 panel), which comprises 60 cancer cell lines from nine different cancer types (breast, central nervous system, colon, kidney, leukemia, lung, melanoma, ovary and prostate). The data were downloaded from CellMiner and based on an integration of five different microarray platforms (5-Platform, Affymetrix HG-U95, HG-U133, HG-U133 Plus 2.0, GH Exon 1.0 ST, and Agilent WHG) yielding a z-score for each gene of each sample (details, see [26]). Missing values were replaced by the mean expression values of the according genes. The cell line SF 539 was excluded from our analysis because of a large number (*N* = 10,404) of undefined entries. Subsequently, we continued the analysis of *MITF*’s TFs on a second, independent dataset, to see whether our findings are consistent and reproducible. Therefore, we used gene expression data from melanoma cells taken from a study by Hoek et al. [16, 17]. In brief, melanoma cells were released from tissue sections of melanoma metastases. Cells were cultured, total RNA was extracted, labeled and their transcriptome profiled using Affymetrix HG-U133 plus 2.0 oligonucleotide microarrays. Raw intensity signals were normalized employing Affymetrix MAS 5.0. Values below 0.01 were set to 0.01 and each value was divided by the 50^th^ percentile of all values in that sample. Each expression value was divided by the median of its values in all samples. Finally, expression values were z-normalized for each gene. For our analysis, we used expression data from 33 samples from the Mannheim cohort of the study by Hoek and coworkers (subsequently denoted as the Mannheim cohort). Cell lines from this panel were also used for our in vitro experiments. For inferring clinical and expression data, we used skin cutaneous melanoma (SKCM) samples from the Cancer Genome Atlas (TCGA; http://cancergenome.nih.gov/). Clinical as well as *MITF*, *SOX5* and *SOX10* mRNA expression (RNA Seq V2 RSEM) data were downloaded from the cBio portal (http://www.cbioportal.org/). The SKCM expression data were z-normalized. For the comparison of expression levels between non-survived and survived subgroups Wilcoxon rank sum tests were applied, because the distribution of the expression levels was not normally distributed. All data sets used are publically available.

### Cell culture

Five melanoma cell lines used in the Hoek and coworkers analysis [16, 17] MaMel-122, MaMel-86b, MaMel-61e and MaMel-79b (own laboratory) as well as A375 purchased from ATCC were cultured at 37 °C and 5 % CO_2_ in RPMI 1640 medium (Gibco, Carlsbad, CA, USA) + 10 % FCS in general without antibiotics. MaMel-122-pMITF-GFP was cultured in medium containing 0.5 μg/ml puromycin (Sigma-Aldrich, Steinheim, Germany). These cell lines were chosen because they exhibit substantial expression of MITF, SOX5 and SOX10.

### siRNA transfection and qRT-PCR

To investigate the effects of SOX5 and SOX10 on *MITF* expression levels, Ambion® Silencer® Select Pre-designed (Inventoried) siRNAs (Life Technologies, Carlsbad, CA, USA) were utilized to knock down these transcription factors. For the knock-down of SOX5 or SOX10 siRNA **s13303** (Antisense sequence, no overhangs**: UCCUUUCACACCGUAAGUG**) and siRNA s13308 (Antisense, no overhangs: **UCCUUCUUCAGAUCGGGCU**) were used, respectively.

For validation of siRNA mediated knock-down effects and to diminish off-target effects, defined, high complexity SOX5 and control siRNA pools consisting of 30 individual siRNAs each (siTOOLs Biotech, Planegg, Germany) [27] were included. Melanoma cells were seeded in 12-well plates and cultured for 24 h to reach 70–80 % confluency. Transfections were performed according to the manufacturer’s instructions (DharmaFect transfection reagent; GE Healthcare, Little Chalfont, United Kingdom) using 25 nM single siRNAs and 10 nM for siRNA pools. Forty eight h post transfection, cell pellets were collected and stored at -80 °C until RNA was isolated with the miRNeasy Mini Kit (Qiagen, Hilden, Germany) according to the manufacturer’s protocol. Reverse transcription was performed using the Transcriptor First Strand cDNA Synthesis Kit (Roche Applied Science, Mannheim, Germany). 500 ng of total RNA was reverse transcribed in a 20 μl reaction utilizing oligo(dT) primers. The cDNA was diluted (1:5) with PCR-quality water (Sigma-Aldrich, Steinheim, Germany) and 2 μl of the cDNA dilution was used for qRT-PCR in a 20 μl reaction using the TaqMan® Universal PCR Mastermix (Applied Biosystems, Foster City, CA, USA). The qRT-PCR was performed for *MITF, SOX5* or *SOX10* as the gene of interest (GOI) and glyceraldehyde-3-phosphate dehydrogenase (*GAPDH*) as the housekeeping gene (HK) using the TaqMan probes HS01117294_m1 MITF, HS00753050_s1 SOX5, HS00366918_m1 SOX10 (Life Technologies, Carlsbad, CA, USA) and HuGAPDH (Applied Biosystems, Foster City, CA, USA), respectively. The qRT-PCRs were run on an Applied Biosystems 7300 Real Time PCR system. For all samples, three technical replicates were performed for both *MITF* and *GAPDH*. Median Ct values for *MITF* and *GAPDH* were calculated based on three technical replicates for the different samples. ∆Ct values were calculated according to

5 $$ \varDelta Ct=C{t}_{MITF}-C{t}_{GAPDH} $$

to normalize the *MITF* level to the control (*GAPDH).* ∆∆Ct values were calculated according to

6 $$ \varDelta \varDelta Ct=C{t}_{transfected}-C{t}_{control} $$

to normalize the sample transfected with siRNA against *SOX5* or *SOX10* mRNA to the control condition. Finally, the fold change was calculated according to

7 $$ Foldchange={2}^{-\varDelta \varDelta Ct} $$

### *MITF*-promoter reporter assay

Stable transfection of MaMel-122 cells was performed to generate a cell line that expresses the green fluorescence protein (GFP) gene downstream of the *MITF* promoter.

The human *MITF* promoter was amplified from the plasmid pMI, kindly provided by Dr. Ballotti [28], with the following primers:

- *MITF* prom forward → CGCATCGATAGGCCGTTAGAAACATGATC
- *MITF* prom reverse → CGCTCTAGACAATCCAGTGAGAGACGGTAG

The amplified promoter was cloned into pLenti CMV GFP Puro (pLenti CMV GFP Puro (658-5) was a gift from Eric Campeau; Addgene plasmid # 17448) [29]. For this purpose, the CMV promoter was cut from pLenti CMV GFP Puro with ClaI and XbaI and the *MITF* promoter was introduced at the same position. A plasmid map of the used vector MITFP-pLenti can be found in the supplement (Additional file 1: Figure S2). Functional validation of the vector was performed in primary human melanocytes in comparison to human fibroblasts (Additional file 1: Figure S3). Melanocytes and fibroblasts were isolated following standard protocols from skin remainings after operations such as foreskins after circumcisions of healthy donors. Successfully MITFP-pLenti transfected cells were positively selected using 0.5 μg/ml puromycin.

The generated cell line was denoted by MaMel-122-pMITF and was constantly kept under selective pressure. MaMel-122-pMITF was used to investigate the role of SOX10 and SOX5 in regulating *MITF* at the transcriptional level. siRNA transfection experiments were performed analogous to the qRT-PCR analyses. 1 · 10^5^ cells were seeded in 24-well plates and cultured for 24 h. Then, the wells were transfected with either SOX10 siRNA, SOX5 siRNA, non-targeting control siRNA or a mixture of both, i.e. SOX10 and SOX5 siRNA. The final concentration of each siRNA per well was 25 nM. The cells were harvested 72 h after transfection. The cells were detached from each well with 50 μl trypsin and resuspended in 150 μl of medium. After centrifugation, the cell pellets were washed once with 200 μl PBS and three times with 1 ml ice cold FACS buffer. Finally, the pellets were dissolved in 200 μl FACS-buffer and fluorescence measurements were performed using a BD FACSCalibur^TM^ (BD Biosciences) flow cytometer using channel FL-1 to detect GFP. Unstained MaMel-122 cells were included in each individual measurement as a negative control. The analysis of the flow cytometry data was conducted using FlowJo version 9.6.4 (http://www.flowjo.com/).

### Proliferation assay

The effect of SOX5 on cell viability was assessed using CellTiter-Glo Luminescent Cell Viability Assay (Promega, Fitchburg, WI, USA) after transfection with 10 nM control or SOX5 siRNA pools. 1 x 10^4^ cells (fast growing) and 2 x 10^4^ cells (slow growing) cells were seeded per well in 96 well black/clear flat bottom plates (Corning, Corning NY). Viability was measured according to the manufactures instructions 24, 48 and 72 h after transfection. Three biological replicates were performed for each condition.

### Invasion assay

Invasion assays were performed 48 h after transfection with SOX5 or control siRNA pool (10 nM) in 24 well plate format. Therefore, 5 x 10^4^ cells resuspended in 50 μl serum-free medium (three technical replicates) were pipetted into the upper insert of a 96 well transwell plate (Corning, Corning NY) coated with 50 ng matrigel/well. The lower chambers were filled with 150 μl medium + 10 % FCS as a chemoattractant. After 24 h, invaded cells were detached from the membrane, washed, stained with calcein AM (Thermo Fisher Scientific, Waltham, MA) and analyzed with a fluorometer according to the manufactures protocol.

### Statistical analysis

Statistical significance was calculated using the one-sided two-sample Student’s *t*-test and a Wilcoxon rank sum test was performed for non-normally distributed populations (comparing the distribution of *SOX5* expression between different subgroups of SKCM data (survived vs. non-survived, thin vs. thick)). *P*-values less than 0.05 were considered statistically significant. For the Kaplan-Meier analysis the cutoffs for low and high expression were determined by a 10-fold cross-validation approach using the R-package maxstat [30]. The median cutoff was used to classify samples into low and high expression subgroups and nonparametric log-rank tests were used to assess significance. All statistical analyses were performed using R version 3.0.1 (http://www.r-project.org/) and Microsoft Excel 2013.

### Prediction of Breslow thickness

A linear model consisting of the expression for the three transcription factors SOX5, MITF and SOX10 was optimized by minimizing the differences between measured Breslow thickness *t*_*j*_ for sample *j* and predicted Breslow thickness $\widetilde {t_{\mathrm{j}}}$ via minimizing the sum of error terms *e*_*j*_:

8 $$ {\displaystyle {\sum}_{j=1}^l\kern0.5em \mid {t}_j-{\tilde{t}}_j\mid \kern0.5em =}\kern0.5em {\displaystyle {\sum}_{j=1}^l{e}_j} $$

The Breslow thickness was predicted for melanoma patient sample *j* using the linear model

9 $$ {t}_j={\beta}_0+{\beta}_{SOX5}\cdot ef{f}_{SOX5,j}+{\beta}_{SOX10}\cdot ef{f}_{SOX10,j}+{\beta}_{MITF}\cdot ef{f}_{MITF,j} $$

with *β*_*0*_ as an additive offset, *β*_*TF*_ as the optimization parameter for the TF (SOX5/SOX10/MITF) and *eff*_*TF,j*_ as the estimated effect of a TF in sample *j*. As effect *eff*_*Tf,j*_, we used the gene expression of the TF in sample *j.*

## Results

### The workflow

**Fig. 1:**
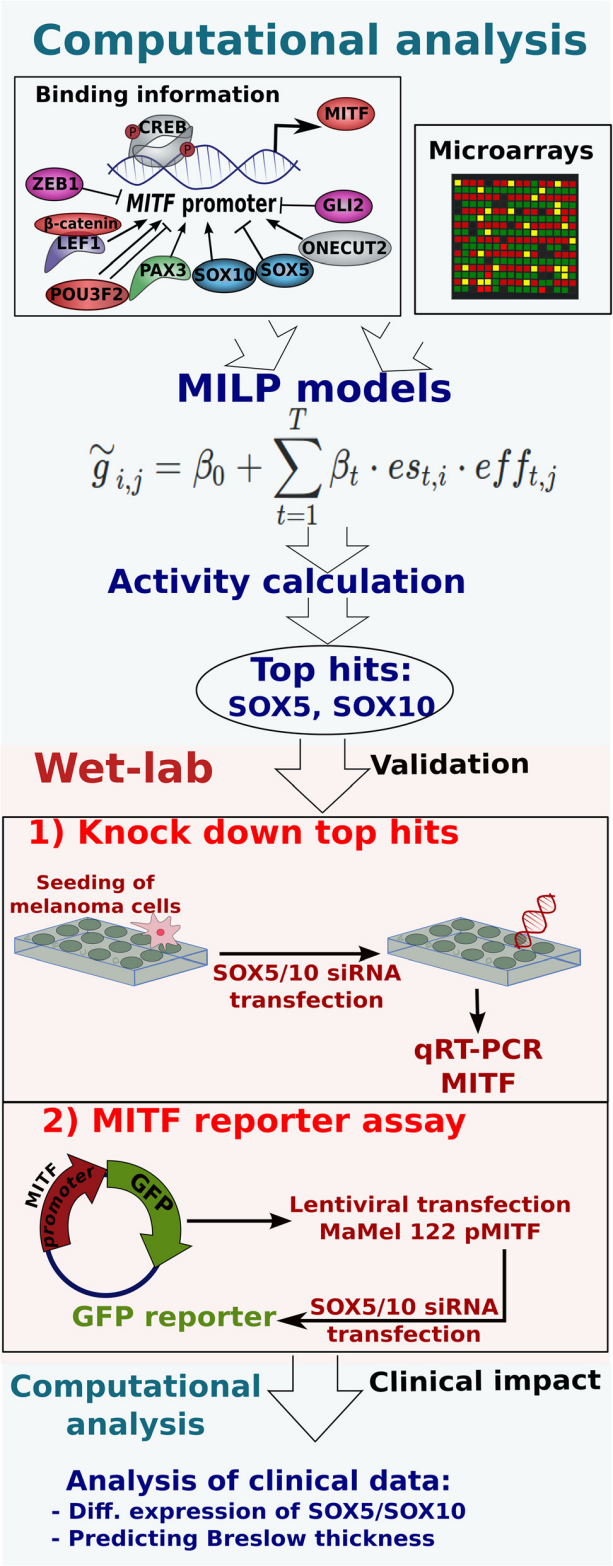
Workflow. We used a regression approach (based on Mixed Integer Linear Programming, MILP) to design a gene regulatory network model. The model aimed to predict *MITF* expression in order to find the regulators that best explain changes in *MITF* expression levels across different cell lines. For this, we used transcription factors known to bind at the promoter of *MITF* extracted from databases and the literature. After this, we performed wet-lab experiments, the effects of the predicted transcription factors (SOX5 and SOX10) were validated using transfection assays with siRNA against these transcription factors and *MITF*-promoter reporter assays. Finally, the clinical impact of MITF and its regulating transcription factors (SOX5, SOX10) was analyzed by investigating expression levels within melanoma tumor samples according to different clinically relevant parameters (non-survival *versus* survival; thin *versus* thick tumors)

The workflow is depicted in Fig. 1. First, we selected all information on transcription factors binding to the *MITF* promoter from several databases and from an analysis of position weight matrices. The resulting transcription factors were the candidates for *MITF* regulation in our investigated cancer cell samples. A regression model was constructed using mixed integer linear programming (MILP). The MILP models were trained with training sets using a calculated sample specific activity of each of the putative transcription factors to predict the transcript levels of *MITF*. The trained models were applied to a validation set and the prediction performance calculated (Pearson correlation between predicted and measured gene expression of each sample). Models were built with an increasing number of included transcription factors in each round, starting with one transcription factor up to all candidate transcription factors. In each round, the MILP model selected the optimal set of regulators. Performing this within several iterations and a cross-validation scheme, the best performing transcription factors were selected (SOX5 and SOX10). Their regulatory effect on *MITF* expression was experimentally validated measuring *MITF* expression of SOX5 and SOX10 knockdowns and by an *MITF* promoter reporter assay. Finally, the clinical implications were investigated by comparing the expression profiles of *SOX5, SOX10* and *MITF* to clinically relevant parameters (overall survival and tumor stage), leading to a biomarker regression model (of SOX5, SOX10 and MITF).

### Identifying the regulators of MITF *in silico*

**Fig. 2:**
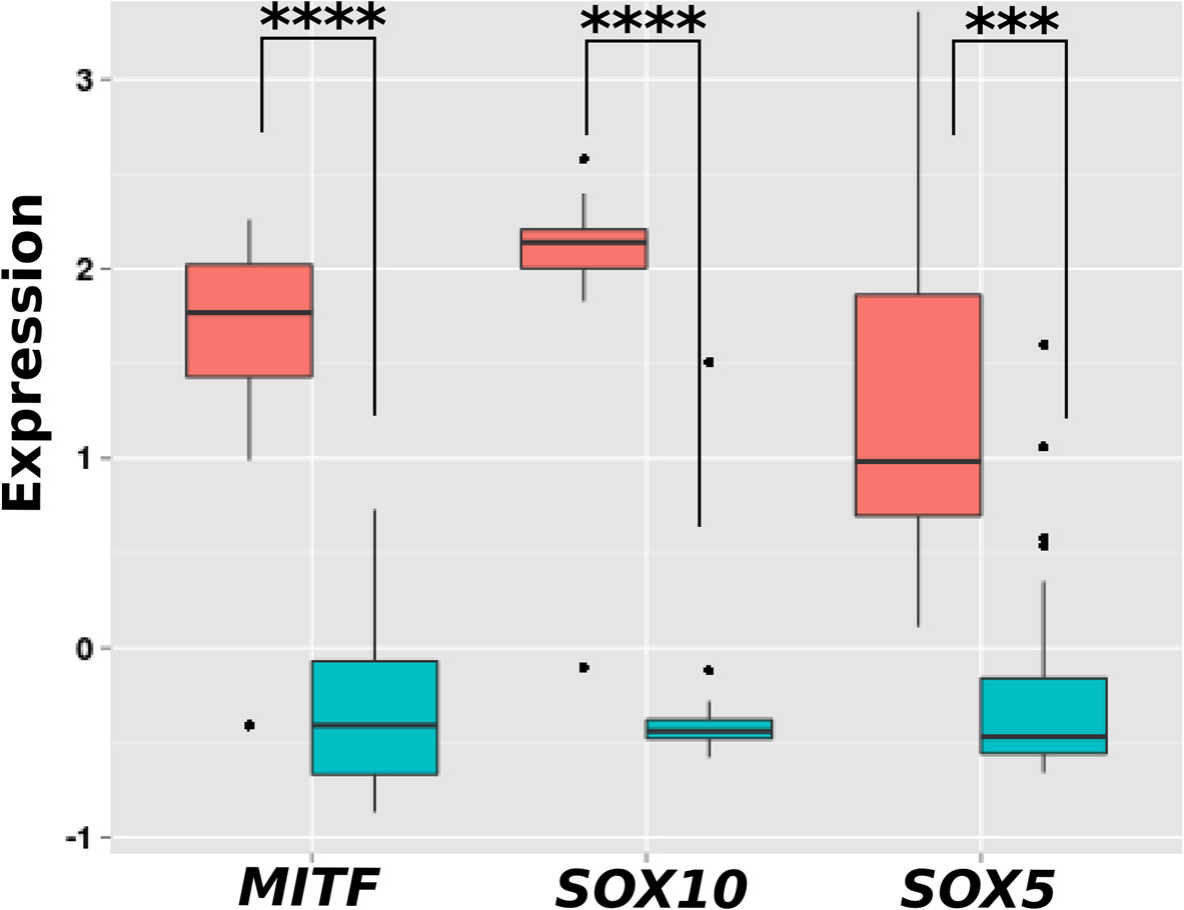
Expression of *SOX5*, *MITF* and *SOX10* in 59 cell lines of the National Cancer Institute (NCI-60 panel). The expression of *SOX5*, *MITF* and *SOX10* was compared between melanoma samples in the NCI-60 panel and the remaining cancer types. All three genes showed significantly higher expression in melanoma cell lines. Statistical significance was determined by two-sided two-sample Student’s t-tests. ***p < 0.001; ****p < 0.0001

The first task was to identify TFs that best explain *MITF* expression. To identify MITF regulators distinctively and differentially active in melanoma cells, we investigated the expression dataset of the NCI-60 panel comprised not only of melanoma cells but also of cells from several other tumor entities. A list of the summarized results of the bottom-up procedure can be found in Table 1. The first regulator selected by the model was SOX5. A model consisting of SOX5 alone had a very good prediction performance with a Pearson correlation of *r* = 0.83 (modeled gene expression of *MITF* versus measured gene expression of the microarrays). In addition, correlation of our activity parameter *act*_*j,t*_ for TF t in cell line *j* with *MITF* expression levels *g*_*j,MITF*_ revealed SOX5 as the top-correlating regulator with Pearson Correlation Coefficient (PCC) *r* = 0.85 (a list of correlations for all investigated TFs is given in Additional file 1: Table S1). Also, SOX10 showed a very good performance in the model (PCC *r* = 0.73) and also its activity correlated very well with *MITF* expression (PCC *r* = 0.73). We wanted to know how well the two transcription factors SOX5 and SOX10, taken together, can explain the expression of MITF. Hence, we constructed a linear regulation model consisting of SOX5 *and* SOX10. This model showed very good performance for predicting *MITF* expression levels for the NCI-60 cell lines with an average PCC of *r* = 0.83. Furthermore, hierarchical cluster analysis (average linkage and Euclidean distance) showed that *SOX5* and *SOX10* expression is sufficient to clearly distinguish melanoma from other cancer types. Nine out of 10 melanoma samples clustered together (Additional file 1: Figure S4). MITF is often referred to as the master regulator of melanocytes and melanoma cells and it is not surprising that it is differentially expressed in melanoma cell lines compared to cells from other cancer types (significance of differential expression of *MITF: p* = 2E-5). Interestingly, also *SOX5* (*p* = 0.0008) and *SOX10* (*p* = 3E-6) showed a significantly higher expression in melanoma samples compared to all other cells (Fig. 2). Furthermore, we wanted to confirm computationally that SOX5 and SOX10 are regulators of *MITF* expression in melanoma cells and thus analyzed a dataset of melanoma cells only. We repeated this analysis with a publicly available melanoma cell line set described by Hoek et al. [16, 17]*.* Consistently, SOX5 and SOX10 exhibited the best correlations of the activity and the gene expression of *MITF* (PCC *r* = 0.75 and *r* = 0.69). Performing the modeling, SOX5 was again the selected TF that could alone predict *MITF* expression best*.* Strikingly, we obtained very good prediction results (PCC *r* = 0.76) by using the optimization parameters of the SOX5/SOX10 model learned on the NCI-60 data for the prediction of *MITF* levels of the independent melanoma data set (33 melanoma samples).

**Table 1:** Results of the bottom-up approach for modeling *MITF* regulation using Mixed Integer Linear Programming

No. of TFs	Predicted TFs	Performance*
1	SOX5	0.83
2	ESR2, SOX5	0.87
3	ESR2, PAX2, SOX5	0.88
4	ESR2, NFKB1.1, PAX2, SOX5	0.89
5	ESR2, NFKB1.1, PAX2, SOX5, ZEB1	0.90
6	ESR2, NFKB1.1, ONECUT2, POU3F2, SOX5, ZEB1	0.91
7	ESR2, NFKB1.1, ONECUT2, PAX2, POU3F2, SOX5, ZEB1	0.91
8	ESR2, GLI2, NFKB1.1, ONECUT2, PAX3, POU3F2, SOX5, ZEB1	0.91
9	ESR2, GLI2, NFKB1.1, ONECUT2, PAX2, PAX3, POU3F2, SOX5, ZEB1	0.90
10	ESR2, GLI2, IRF1, NFKB1.1, ONECUT2, PAX2, PAX3, POU3F2, SOX5, ZEB1	0.92
11	BHLHE40, ESR2, GLI2, IRF1, NFKB1.1, ONECUT2, PAX2, PAX3, POU3F2, SOX5, ZEB1	0.92
12	ESR2, LEF1, NFKB1.1, ONECUT2, PAX2, PAX3, PAX6, PDX1, POU3F2, SOX5, SOX9, ZEB1	0.92
13	ESR2, LEF1, NFKB1.1, ONECUT2, PAX2, PAX3, PAX6, PDX1, POU3F2, SOX5, SOX9, TCF4, ZEB1	0.91
14	BHLHE40, ESR2, LEF1, NFKB1.1, ONECUT2, PAX2, PAX3, PAX6, PDX1, POU3F2, SOX5, SOX9, TCF4, ZEB1	0.91
15	BHLHE40, ESR2, GLI2, LEF1, NFKB1.1, ONECUT2, PAX2, PAX3, PAX6, PDX1, POU3F2, SOX5, SOX9, TCF4, ZEB1	0.90
16	BHLHE40, ESR2, GLI2, LEF1, NFKB1.1, ONECUT2, PAX2, PAX3, PAX6, PDX1, POU3F2, SOX10, SOX5, SOX9, TCF4, ZEB1	0.89
17	BHLHE40, ESR2, GLI2, LEF1, NFKB1.1, ONECUT2, PAX2, PAX3, PAX6, PDX1, POU3F2, SOX10, SOX2, SOX5, SOX9, TCF4, ZEB1	0.89
18	BHLHE40, ESR2, GLI2, IRF1, LEF1, NFKB1.1, ONECUT2, PAX2, PAX3, PAX6, PDX1, POU3F2, SOX10, SOX2, SOX5, SOX9, TCF4, ZEB1	0.87
19	BHLHE40, CREB1, ESR2, GLI2, IRF1, LEF1, NFKB1.1, ONECUT2, PAX2, PAX3, PAX6, PDX1, POU3F2, SOX10, SOX2, SOX5, SOX9, TCF4, ZEB1	0.86

*Averaged Pearson correlation of the model from the training data compared to the validation data

The regulatory network model and the estimated activity values revealed SOX5 and SOX10 as important regulators of *MITF.* In agreement with our findings, SOX10 is a commonly known activating regulator of *MITF* [18, 31] in human. *MITF* regulation by SOX5 has only been shown in murine cells so far [32] and hence we were interested in the regulatory effect of SOX5 on *MITF* in human melanoma cells and tumors, and its regulatory effect in combination with SOX10. Thus, we performed functional assays to validate our *in silico* predictions in human melanoma cells and investigated the expression signatures of MITF, SOX5 and SOX10 in respect to clinically relevant parameters.

### Experimental validation

**Fig. 3:**
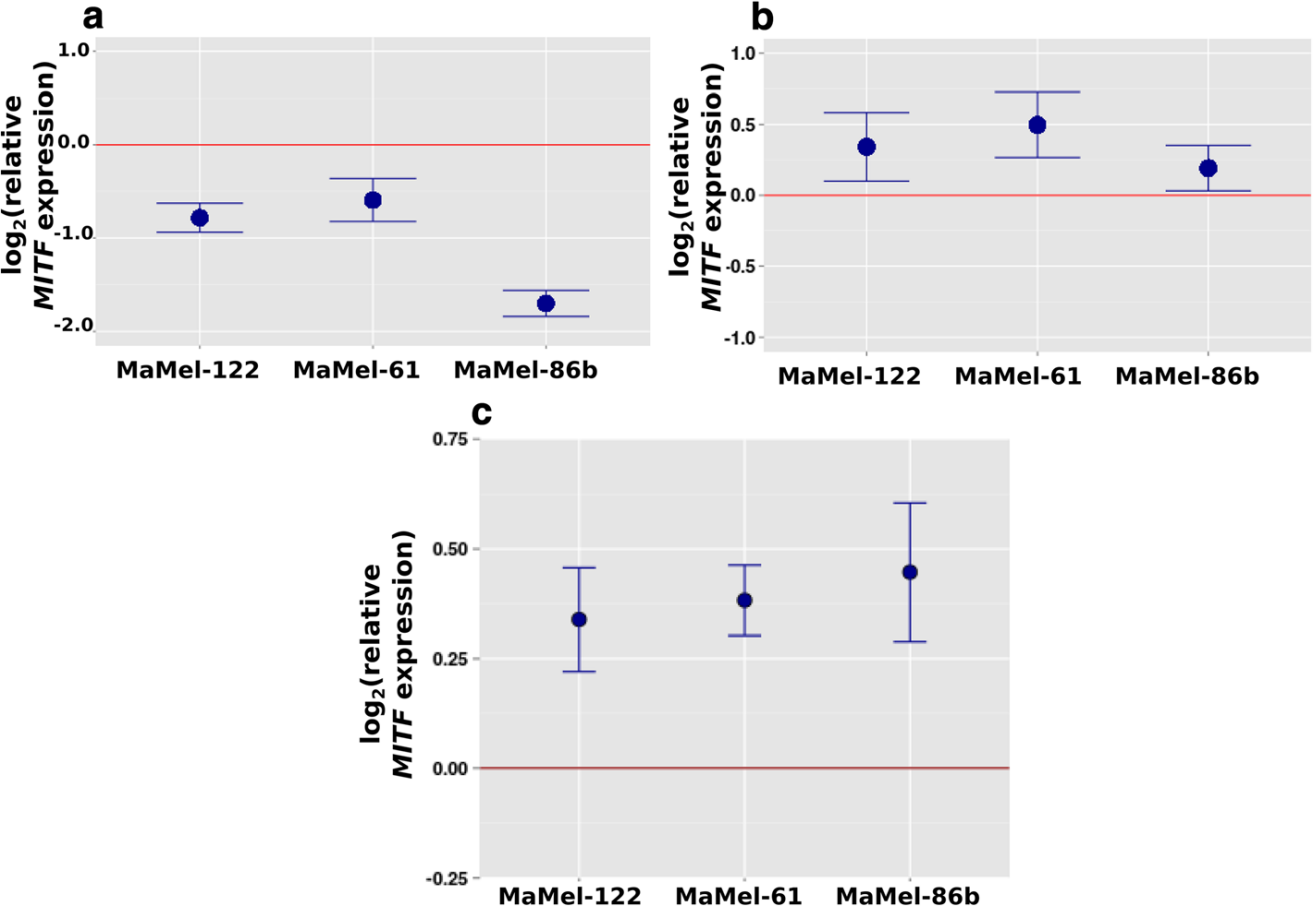
Change in MITF expression 48 h after siRNA transfection. The melanoma cell lines MaMel-122, MaMel-86b and MaMel-61e were transfected with **(a)** 25 nM *SOX5* siRNA s13303 or **(b)** 25 nM *SOX10* siRNA s13308. *MITF* expression was measured by qRT-PCR, normalized to GAPDH expression and control siRNA transfected cells. Graphs show the mean expression and standard deviation of fold changes. Knockdown of *SOX5* resulted in a significant increase in *MITF* expression in all three cell lines, whereas knockdown of *SOX10* led to diminished *MITF* expression. In all three cell lines, the *MITF* expression significantly decreased after *SOX10* knockdown. At least four independent biological replicates were performed for each condition. To verify the effect, the transfections were repeated with SOX5 siRNA pool and control siRNA pool (10 nM) **(c)** with four biological replicates per condition. For all three investigated cell lines, the increase in MITF expression after SOX5 knockdown could be confirmed

SOX10 and SOX5 were individually knocked down by siRNA transfection experiments and significant changes of *MITF* levels were detected by qRT-PCR (Fig. 3). In all three tested melanoma cell lines (MaMel-61e, MaMel-122 and MaMel-86b) the knockdown of SOX5 resulted in significantly increased *MITF* levels compared to cells transfected with control siRNA. In contrast, knockdown of SOX10 resulted in significantly decreased *MITF* levels in all three cell lines. This is in line with the reported observation that SOX10 is an activator of *MITF* [18, 31, 32]. For MaMel-86b the *MITF* level was about 3.5 times lower in the SOX10 siRNA transfected sample compared to the control. To verify the effect of SOX5 knockdown on *MITF* levels, we repeated the transfection experiments with siRNA pools. These pools consisted of 30 individual siRNAs which strongly decrease off-target effects [27]. We estimated the SOX5 and SOX10 knockdown efficiency via qRT-PCR and observed a knock-down efficiency of 50–60 % (Additional file 1: Figure S5). The effect on *MITF* expression levels was confirmed: in all three cell lines, *MITF* levels were significantly increased after SOX5 knockdown compared to control pool transfections (Fig. 3).

**Fig. 4:**
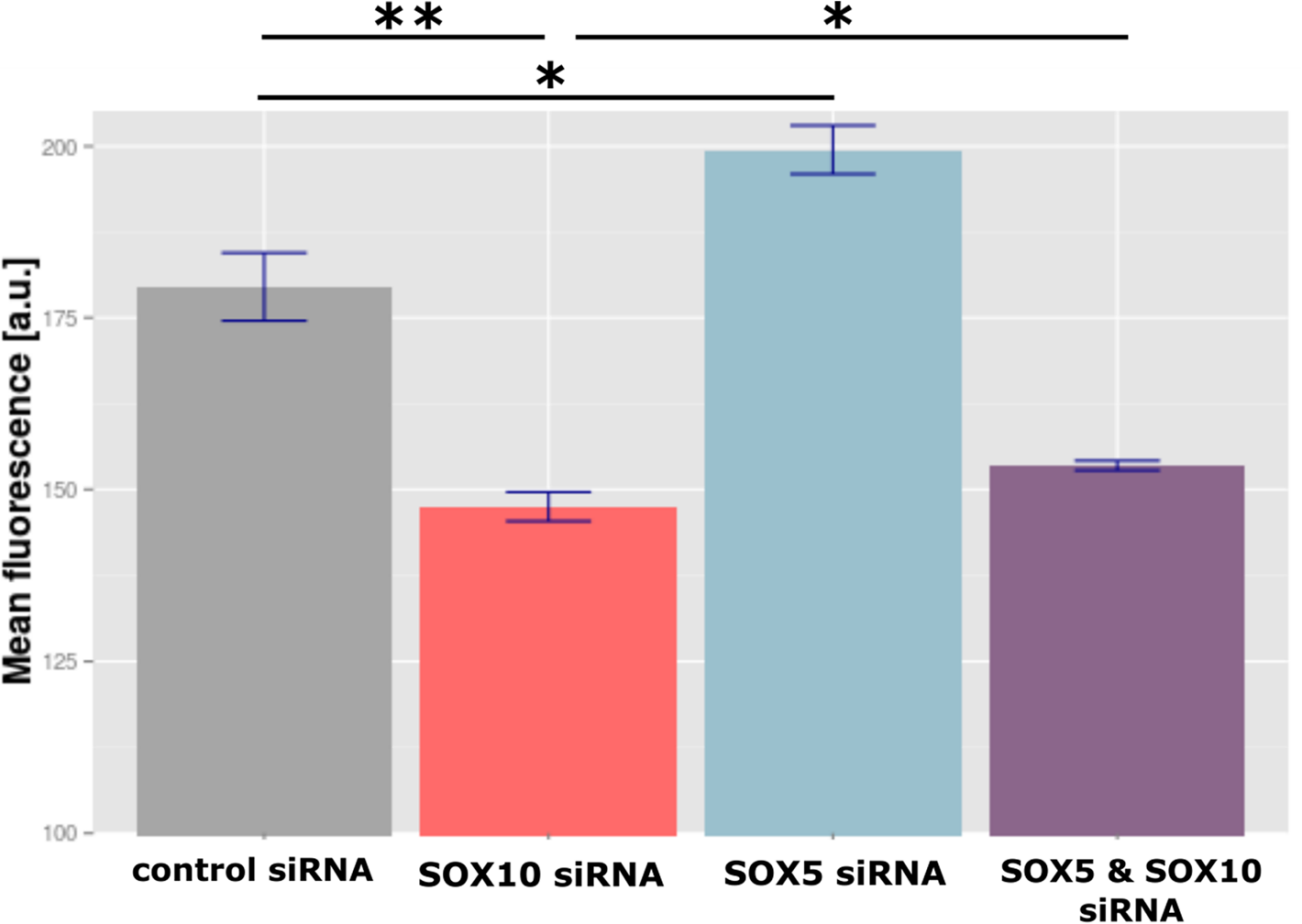
GFP fluorescence of MaMel-122-pMITF cells 72 h post-siRNA transfection. The mean fluorescence of the GFP reporter gene was calculated based on all investigated cell lines. All samples were compared to the control condition and unstained MaMel-122 cells were used as the negative control. For each condition, two biological replicates were performed. Statistical significance was determined by two-sided Student’s t-tests. *p < 0.05; **p < 0.005

We validated these results with a promoter-reporter assay using MaMel-122-pMITF cells, which were stably transfected with a GFP reporter containing an *MITF* promoter. Again, knockdown of SOX10 resulted in a significantly reduced reporter (GFP fluorescence) signal (*p* = 0.007) compared to the control reflecting lowered *MITF* promoter activity. In contrast, knockdown of SOX5 resulted in a significantly increased reporter signal (*p* = 0.022) compared to the control reflecting increased *MITF* promoter activity. Furthermore, a combined knockdown of SOX10 and SOX5 was investigated. As expected, we observed a rescue effect: the reporter signal was increased compared to exclusively knocking down SOX10 (*p* = 0.031) (Fig. 4). In summary, we could confirm our computational predictions, i.e. SOX5 is inducing and SOX10 is repressing *MITF* expression in the observed melanoma cells.

### Phenotypic effects of SOX5 knockdown

To analyze the effects of SOX5 knockdown on viability and invasion we transfected five melanoma cell lines with SOX5 siRNA or control siRNA pools. Viability of SOX5 siRNA transfected cells was assessed 24, 48 and 72 h post transfection and compared to the controls. A decreased proliferation rate was observed in all SOX5 siRNA transfected melanoma cells, except for one cell line (cell line A375, see Table 2, Additional file 1: Figure S6). The effect of SOX5 knockdown on invasion was assessed by the Boyden chamber invasion assay after transfection with SOX5 or control siRNA pools. We observed a reduced invasive behavior in the strongly invading cell lines MaMel-122, MaMel-86b and A375. In contrast, the poorly invading cell lines MaMel-61 and MaMel-79b did not show a reduction in invasion after SOX5 knockdown (see Table 2, Additional file 1: Figure S7).

**Table 2:** Effects of SOX5 siRNA on cell viability and invasion

Cell line	Proliferation			Invasion
	24 h	48 h	72 h	24 h
A375	1.26	1.17	0.99	0.77 (*)
MaMel-79b	0.97	0.91 (*)	0.84 (***)	1.09
MaMel-61e	0.90	0.95	0.79 (***)	1.15
MaMel-122	0.90	0.89 (***)	0.82 (***)	0.87 (*)
MaMel-86b	0.81 (*)	0.82 (*)	0.93	0.62 (*)

Numbers give ratios of SOX5 to control siRNA pool transfected samples. Assays were performed at the indicated time points after transfection. *P-value < 0.05 and ***P-value < 0.0005

### Clinical impact

**Fig. 5:**
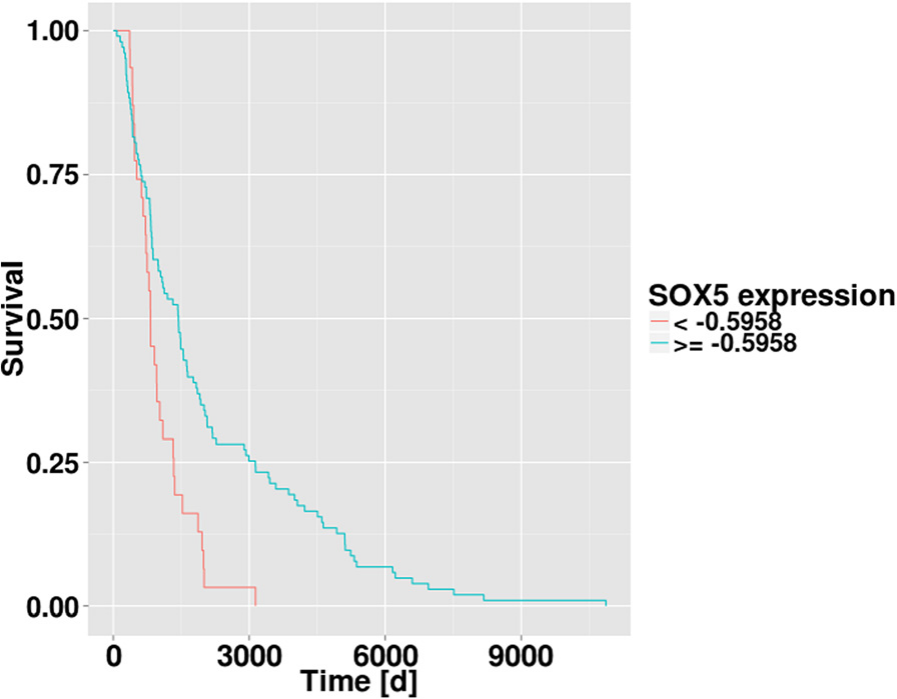
Survival analysis. The SKCM samples were divided based on their *SOX5* expression and based on their survival times (days to death). Kaplan-Meier plot was generated. A significant difference of the two survival distributions could be observed (*p* = 0.0006; log-rank test) with an improved survival rate for the subgroup with higher *SOX5* expression (≥-0.5958)

Having confirmed the involvement of SOX5 and SOX10 in *MITF* regulation experimentally, we wanted to unravel the clinical impact of this regulatory network using the expression data of melanoma tumor samples obtained from The Cancer Genome Atlas (SKCM, Skin Cutaneous Melanoma, http://cancergenome.nih.gov/). Overall, 352 samples of SKCM were used for the analysis. We performed a cross-validation based Kaplan-Meier analysis (see Methods) and determined the optimal cutoff of *SOX5* expression as -0.5958 with better survival for the subgroup with a *SOX5* expression equal or higher than -0.5958 (Fig. 5). The discrimination of patients considering *MITF* or *SOX10* expression did not reveal a significant difference of survival. Furthermore, we compared the expression levels between the subgroups of primary melanoma (69 samples) and distant metastasis (39 samples). Only for *SOX5* expression, a clear tendency (*p* = 0.06) between the subgroups of primary tumor and metastases samples could be observed with a differential *down-regulation* of *SOX5* in primary samples, when compared to metastatic samples (see Additional file 1: Figure S8). In summary, higher *SOX5* expression was associated with a better clinical course of melanoma patients; we thus further studied the clinical relevance of *SOX5* expression investigating the Breslow thickness of primary melanoma tumors.

The Breslow thickness resembles the thickness of the primary tumor of local melanomas and is used to classify melanoma into different tumor stages (T1 - T4). The Breslow thickness is negatively correlated with overall survival and can be used to categorize patients into subgroups [2, 33]. Here, we have analyzed a possible association of Breslow thickness with *SOX5*, *SOX10* and *MITF* expression employing a MILP-based linear modeling approach. To test for such an association, we investigated, if *SOX5, SOX10* and *MITF* can be used to predict Breslow thickness. The prediction was performed for 266 samples from the SKCM data set with available Breslow thickness and gene expression data. However, the estimated prediction performance (based on a leave one out cross validation for all samples) was very poor (PCC *r* = 0.02). Previous studies showed that the Breslow thickness provides more prognostic information if cut points are used [34]. In line, we divided the samples into subsets according to their Breslow thickness used for the classification of the tumor staging (T1-T4). This improved the performance considerably, in particular for the group of tumors with thin thickness (Table 3). Good or reduced prediction performance was obtained for melanoma with thin (<=1 mm; *r* = 0.53, *n* = 39) and intermediate thickness (>1 and <4 mm; *r* = 0.24; *n* = 125), respectively. In contrast, prediction for thick tumors showed no significant correlation with thickness (>4 mm; *r* = 0.07; *n* = 102). As expected, survival times of our investigated tumor samples with a small Breslow thickness (<1 mm) was significantly higher (*p* = 0.005) than the remaining samples.

**Table 3:** Prediction of Breslow thickness for SKCM melanoma samples using the regression model of SOX5/MITF/SOX10

Group	Thickness	Number of samples	PCC r*
All samples	-	266	0.02
Thin	< 1 mm	39	0.53
Intermediate	1 – 4 mm	125	0.24
Thick	> 4 mm	102	0.07

*Pearson correlation of the model from the training data compared to the validation data

**Fig. 6:**
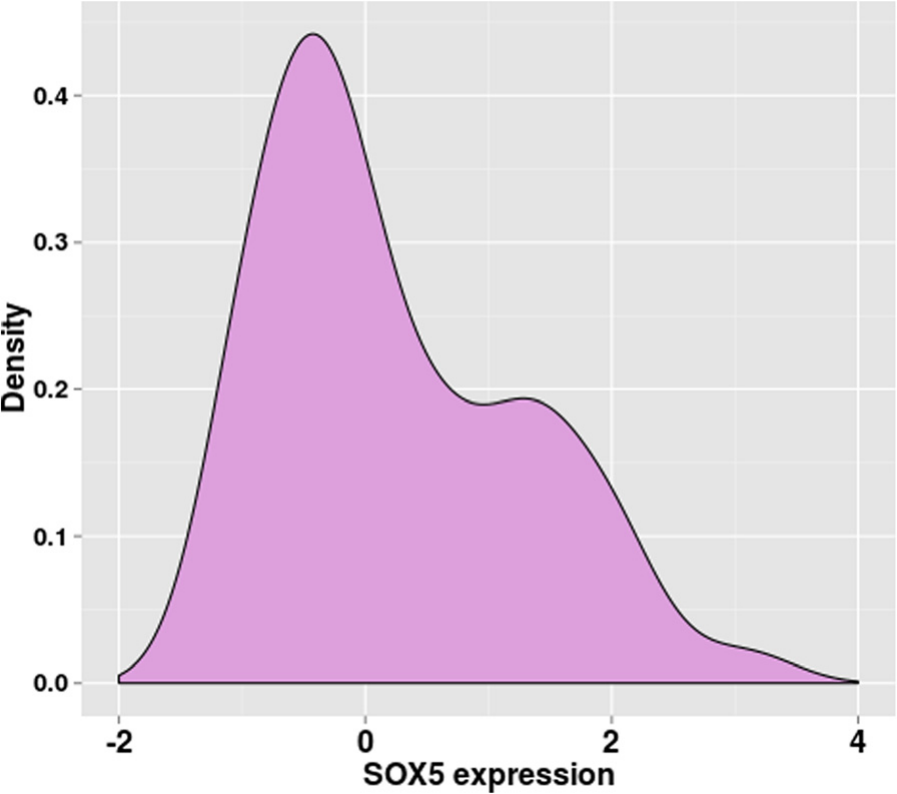
Distribution of *SOX5* expression in the SKCM dataset with vital status dead

To understand the observed differences in prediction performance between subgroups with thin and thick tumors, we investigated the expression distributions of *SOX5, SOX10* and *MITF*. Interestingly, we observed a bimodal distribution of *SOX5* expression. Figure 6 shows the density function of *SOX5* expression of all investigated SKCM samples with the vital status dead pointing to a bivalent role of SOX5. The corresponding histogram and the density function for the subgroup with thick tumors is presented in Additional file 1: Figures S9 and S10, respectively. Also for the investigated 33 melanoma cell lines from the Mannheim cohort [17] which only included samples from stage III and IV melanoma patients, *SOX5* expression followed a bimodal distribution, as shown in Additional file 1: Figure S11.

High percentages of melanoma tumor cells show mutations in the BRAF locus [35] Due to the fact, that in melanoma hyper-activated BRAF often suppresses MITF [13–17], we compared the BRAF mutation status with the expression of *MITF, SOX10* and *SOX5*. Investigating the SKCM dataset, *SOX10* and *MITF* expression tended to be higher in melanoma samples with normal BRAF (*p* = 0.06 and *p* = 0.08, two sided Student’s *T*-test). Strikingly, *SOX5* was significantly overexpressed in samples with mutated BRAF (*p* = 0.006). We observed a weakly positive correlation of SOX5 and MITF expression in the BRAF wildtype subgroup (Pearson’s correlation *r* = 0.18), and weakly negative correlation in the BRAF mutated subgroup (PCC *r* = -0.13) hinting for a stronger regulatory involvement of SOX5 on *MITF* expression in the tumor cells with BRAF mutation. Comparing tumor subgroups of NRAS mutated with NRAS wildtype, no significant expression differences were found except for SOX5 which was significantly overexpressed in NRAS mutated samples (*p* = 0.05). Taken together, SOX5, SOX10 and MITF seem to have a crucial clinical impact and our developed linear regression based expression signature of these three genes associated in particular with melanomas with a small Breslow thickness.

## Discussion

In this study, a transcription factor network was constructed based on chromatin immunoprecipitation binding data from several data repositories and a motif analysis. Using our established regression model (MILP model) and the defined activity, we found the transcription factors SOX5 and SOX10 with which the model could predict best the gene expression values of *MITF* in various melanoma cell lines. Indeed, both transcription factors were capable to explain the differences in *MITF* expression levels when trained with a dataset of cells from different tumors and applied to a different dataset, i.e. a dataset of melanoma cell lines. In particular, SOX5 was found to be a very informative predictor, exhibiting the highest correlation of its calculated activity with *MITF* expression. We confirmed experimentally that SOX5 and SOX10 have an effect on *MITF* expression levels in melanoma cell lines; SOX5 down-regulation increases *MITF* expression, hinting at an inhibitory effect, while vice versa, SOX10 down-regulation led to MITF up-regulation. In addition, our model predicted a combined regulation in which *MITF* transcription is activated by SOX10 and inhibited by SOX5. In line with this, after investigating the expression profiles of the melanoma cell lines (Mannheim cohort of [17]), we observed a correlation between *SOX5* and *SOX10* expression (PCC *r* = 0.43) and an even stronger correlation between the activity of SOX5 and SOX10 (PCC *r* = 0.75). SOX10 is a well-known transcriptional activator of *MITF* [18, 31]. In addition to this, we found SOX5 to be a novel regulator of *MITF* in human melanoma cells. Stolt and coworkers found the involvement of SOX5 in melanocyte development by altering SOX10 activity in mouse models. In mice, SOX5 and SOX10 can bind to the same locus on their target genes *Mitf* and *Dct*. It was shown in B16 mouse melanoma cells that SOX5 prevents the activation of these target genes through site competition with SOX10 [32, 36]. We observed a similar effect in human melanoma cells: A double knockdown of SOX5 and SOX10 partially rescued *MITF* expression compared to a single knockdown of SOX10. We assume that SOX5 regulates MITF via direct binding to the MITF promoter as (i) Stolt and coworkers showed in mice [32], and (ii) as we observed a strong binding profile of the sequence motif in SOX5 to the MITF promoter (see Additional file 1: Figure S1 in the supplementary material); however direct binding remains to be shown with e.g. ChIP experiments.

In addition to SOX5 and SOX10, to a lesser extent, also SOX2 and SOX9 were among our predicted candidates of selected transcription factors (in the models with 12 or more predicted regulators, see Table 1). It is known, that also other SOX family members are involved in melanocyte development [36]. Shakova and coworkers observed an efficient reduction of tumorigenesis in animal models and in human melanoma cells when reducing SOX10 expression levels and for this anti-tumoric effect they found SOX9 to be required as a functional antagonistic regulator of SOX10 [37]. Besides this, Liu and Lefebvre found that the regulatory trio of SOX9, SOX5 and SOX6 cooperatively work together to activate super-enhancers in a genome-wide way in rat chondrosarcoma cells [38]. Taken together, these observations are in line with our observation that SOX5 and SOX10 have an opposing effect on regulation of the central transcription factor MITF. When investigating the SKCM dataset, we found SOX9, SOX2 as well as SOX6 to be down-regulated in the tumor subgroup of low *SOX5* expression compared to the tumor subgroup of high *SOX5* expression (see Additional file 1: Table S2). For the future, it could be intriguing to disentangle the fine grained interplay between these SOX family members and their involvement in tumor progression. The analysis of clinical tumor data (SKCM) revealed that higher *SOX5* expression was a significant indicator for longer survival (Fig. 5). Accordingly, we observed a tendency towards longer survival of patients with tumors showing lower expression of MITF (Additional file 1: Figure S12).

We observed a higher SOX5 expression in metastatic melanoma compared to primary melanoma (Additional file 1: Figure S8), although the survival analysis revealed that very low SOX5 expression is associated with poor prognosis (Fig. 5). This might point towards a dual functional role of SOX5 depending on primary versus metastatic tumor stage. We speculate that SOX5 could be an important factor during the transition from primary to metastatic melanoma, as SOX5 knockdown resulted in reduced invasion (Table 2). As only ten primary melanoma samples from patients who succumbed to disease were available, we performed a correlation analysis on SOX5 expression and survival time resulting in a strongly negative (*r* = -0.65) correlation, whereas in metastatic melanoma samples only a weak correlation could be observed (*r* = -0.12; not shown). This is in line with Riker et al.*,* who observed in their analyses that SOX5 expression is strongly increased in thick versus intermediate melanoma samples (Breslow’s thickness), associated with onset of metastatic phenotype [39]. In contrast, our survival analysis revealed a worse prognosis for patients with tumors expressing low-level of SOX5. Notably, this analysis included mainly metastatic tumor samples and only ten samples from primary tumors. We speculate that in metastatic melanoma the anti-proliferative effect of very low SOX5 and thus high MITF levels might lead to a diminished susceptibility to chemotherapy and thus to a worse prognosis.

Regulation of *MITF* expression is highly complex and mediated by various activating and inhibiting intra- and extracellular processes. Although high MITF levels have an anti-proliferative effect, *MITF* expression is detectable in almost all melanoma tumors. It seems that a basal level of *MITF* expression is necessary for melanoma cells and therefore *MITF* expression and activity is not entirely down-regulated, which is in line with the observation that almost all melanoma cells maintain their ability to synthesize melanin. Wellbrock and coworkers proposed that a low basal MITF level could be important for the survival of melanoma cells and also for their proliferation through regulation of cyclin-dependent kinase 2 (CDK2) and B-cell CLL/lymphoma 2 (BCL2) [40]. They proposed that an intermediate, well-balanced MITF level is important for melanoma cells to survive and proliferate. We add to this the notion that a well-tuned interplay of SOX5 and SOX10 could be crucial for this homeostasis of MITF expression, avoiding too high as well as too low *MITF* expression.

We found that up-regulation of *SOX5* expression co-occurs with BRAF mutations. It might be favorable for the tumor to suppress MITF expression with different strategies like increased BRAF activity that leads to MITF degradation, or increased inhibition of *MITF* transcription due to SOX5 blocking the binding site of SOX10. In future studies, it would be interesting to investigate whether increased *SOX5* expression is a downstream effect of BRAF mutation or whether it is rather an independent control mechanism for *MITF* regulation. Interestingly, the prediction of Breslow thickness using all of the investigated regulators (SOX5, SOX10, and MITF) showed good prediction performance only for thin melanoma tumors (<1 mm) and was rather poor for thick melanomas. This may indicate a transition point in melanoma progression. Indeed, Riker and coworkers reported of a transition point of melanoma progression; they observed that most genes up-regulated in more advanced melanoma exhibit the highest change of their expression level during the transition of intermediate to thick lesions [39]. We also observed a similar transition by modeling Breslow thickness with the three TFs, SOX5, SOX10 and MITF. Interestingly, we identified a bimodal distribution of *SOX5* expression in tumor samples and also in the melanoma cell lines. Cells of a potential subset of melanoma, which is indicated by the bimodal distribution, may use the up-regulation of SOX5 to repress *MITF* in order to prevent its inhibitory effect on proliferation.

## Conclusions

To conclude, we applied a computational approach to infer transcriptional regulation of *MITF* in human melanoma cells employing microarray expression profiles. Besides SOX10, we identified SOX5 regulating MITF in human melanoma cells and validated its inhibitory effect experimentally by functional and reporter assays. We found low *SOX5* expression to be an indicator for shorter survival of patients with melanoma tumors. In the future, SOX5 might play an important role when entangling the fine grained interplay of MITF regulation and its impact on tumorigenesis. SOX5 may suit as a prognostic marker in combination with other biomarkers involved in regulation of MITF.

### Ethics approval and consent

TCGA data used in this study are publically available. Melanoma cell lines were generated at the DKFZ Skin Cancer Unit with the approval by the Ethics Committee II of Heidelberg University and have been published previously [17]. Melanocytes and fibroblasts used to test MITFP-Lenti vector were obtained from healthy donors and this study was approved by the Ethics Committee II of Heidelberg University (approval number: 2009-350 N-MA) and written informed consent was obtained from donors or their parents, if donors were under the age of 16 years.

## Availability of data and materials

All used data sets are publically available. For the SKCM samples expression and clinical data were obtained from TCGA. The clinical data was downloaded from cancergenome.nih.gov and the expression data was downloaded from cbioportal.org [41, 42]. NCI-60 data were obtained from CellMiner [26]. Cell line expression data from Hoek study (Mannheim cohort) can be accessed via NCBIs Gene Expression Omnibus (http://www.ncbi.nlm.nih.gov/geo/) with GEO Series accession GSE4845 [17, 18].

## Additional files

Additional file 1: Table S1. Correlation of activity parameter for all putative MITF TFs with *MITF* expression levels for the NCI-60 panel. **Table S2.** Comparison of expression levels of SOX5 to different SOX family members (from the SKCM samples). **Figure S1.** Shows density plot of TBA z-scores for all TFs and target genes. **Figure S2.** Shows map of the used lentiviral vector MITFP-pLenti. **Figure S3.** Validation of the functionality of the melanocyte-specific lentiviral reporter vector MITFP-pLenti by comparing melanocytes and fibroblasts. **Figure S4.** Hierarchical cluster analysis of the NCI-60 cell line panel based on SOX10, SOX5 and MITF expression. **Figure S5.** Validation of siRNA mediated knock-down of SOX5 by qRT-PCR. **Figure S6.** Results of cell viability assay after transfection with SOX5 siRNA pool for five melanoma cell lines. **Figure S7.** Results of invasion assay after transfection with SOX5 siRNA pool. **Figure S8.** Boxplot *SOX5* expression for primary and distant metastasis samples. **Figure S9.** Histogram of *SOX5* expression for SCKM samples with vital status dead. **Figure S10.** Bimodal distribution of *SOX5* expression for thick subgroup of the SKCM samples with Breslow thickness > 4 mm. **Figure S11.** Density and histogram of *SOX5* expression for melanoma samples from the 33 melanoma cell lines. **Figure S12.** Kaplan-Meier analysis of SKCM samples based on *MITF* expression. (DOCX 1173 kb)
