# Supplementary material for: SOX5 is involved in balanced MITF regulation in human melanoma cells
Source: BMC Med Genomics. 2016 Feb 29;9:10. doi: 10.1186/s12920-016-0170-0 (PMC4772287; doi:10.1186/s12920-016-0170-0)
Supplement: Additional file 1: Table S1. — Correlation of activity parameter for all putative MITF TFs with MITF expression levels for the NCI-60 panel. Table S2. Comparison of expression levels of SOX5 to different SOX family members (from the SKCM samples). Figure S1. Shows density plot of TBA z-scores for all TFs and target genes. Figure S2. Shows map of the used lentiviral vector MITFP-pLenti. Figure S3. Validation of the functionality of the melanocyte-specific lentiviral reporter vector MITFP-pLenti by comparing melanocytes and fibroblasts. Figure S4. Hierarchical cluster analysis of the NCI-60 cell line panel based on SOX10, SOX5 and MITF expression. Figure S5. Validation of siRNA mediated knock-down of SOX5 by qRT-PCR. Figure S6. Results of cell viability assay after transfection with SOX5 siRNA pool for five melanoma cell lines. Figure S7. Results of invasion assay after transfection with SOX5 siRNA pool. Figure S8. Boxplot SOX5 expression for primary and distant metastasis samples. Figure S9. Histogram of SOX5 expression for SCKM samples with vital status dead. Figure S10. Bimodal distribution of SOX5 expression for thick subgroup of the SKCM samples with Breslow thickness > 4 mm. Figure S11. Density and histogram of SOX5 expression for melanoma samples from the 33 melanoma cell lines. Figure S12. Kaplan-Meier analysis of SKCM samples based on MITF expression. (DOCX 1173 kb) [file 12920_2016_170_MOESM1_ESM.docx]

**SOX5 is involved in balanced *MITF* regulation in human melanoma cells**

Theresa Kordaß^1,2^, Claudia E. M. Weber^1^, Marcus Oswald^2,3^, Volker Ast^2,3^, Mathias Bernhardt^4,5^, Daniel Novak^4,5^, Jochen Utikal^4,5^, Stefan B. Eichmüller^1,+^, Rainer König^2,3,6,+,*^

^1^ GMP & T Cell Therapy Unit, German Cancer Research Center (DKFZ), INF 280, 69120 Heidelberg, Germany

^2^ Integrated Research and Treatment Center, Center for Sepsis Control and Care (CSCC), Jena University Hospital, D-07747 Jena, Erlanger Allee 101

^3^ Network Modeling, Leibniz Institute for Natural Product Research and Infection Biology - Hans Knöll Institute Jena, Beutenbergstrasse 11a, 07745 Jena

^4^ Skin Cancer Unit, German Cancer Research Center (DKFZ), INF 280, 69120 Heidelberg, Germany

^5^ Department of Dermatology, Venereology and Allergology, University Medical Center Mannheim, Ruprecht-Karl University of Heidelberg, Mannheim, Germany.

^6^ Theoretical Bioinformatics, German Cancer Research Center, INF 580, 69121 Heidelberg, Germany

**Supplementary Material**

**Table S1** **Correlation of activity parameter act_j,t_ for TF t in cell line j with *MITF* expression levels *g_j,MITF_* for the NCI-60 panel.**

| **Transcription factor** | **PCC r*** |
| --- | --- |
| SOX5 | 0.85 |
| SOX10 | 0.73 |
| SOX9 | 0.69 |
| POU3F2 | 0.67 |
| SOX2 | 0.30 |
| ONECUT2 | 0.10 |
| PAX3 | 0.06 |
| BHLHE40 | 0.00 |
| GLI2 | -0.03 |
| IRF1 | -0.07 |
| TCF4 | -0.11 |
| PDX1 | -0.13 |
| PAX6 | -0.14 |
| LEF1 | -0.18 |
| ESR2 | -0.20 |
| NFKB1,NFKB2,REL,RELA,RELB  (complex) | -0.22 |
| CREB1 | -0.28 |
| PAX2 | -0.40 |
| ZEB1 | -0.63 |

* Pearson Correlation of TF's activity and MITF expression levels

**Table S2** **Comparison of expression levels of SOX5 to different SOX family members** (from the SKCM samples).

| **SOX member** | **SOX5 low^*^** | **SOX5 high^*^** | **P-value^**^** |
| --- | --- | --- | --- |
| SOX10 | 0.57 | 0.29 | 0.18 |
| SOX9 | 0.00 | 0.17 | 0.25 |
| SOX2 | -0.10 | 0.45 | 0.003 |
| SOX6 | -0.23 | 0.08 | 0.05 |

**^*^** Cutoffs for SOX5 expression were set to 20% quantile (z-score=-0.78) and 80% quantile (z-score = 0.72), respectively.

^*^**^*^**P values were calculated with two-sided t-tests. The expression levels of SOX family members were compared between the subgroups of SOX5 high versus SOX5 low expression.


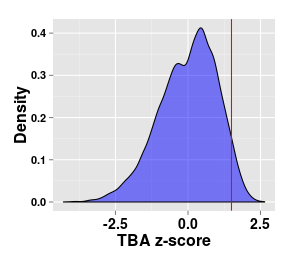
**Fig S1 Density plot of TBA z-scores for all TFs and target genes.** The total binding affinity score of SOX5 binding to human MITF promoter was 1.5. In order to show the significance of this score we plotted all z-scores of the TBA matrix (130 TFs and 22,102 target genes). The Affinity score of SOX5 to the promoter of MITF is marked with a red line.

**
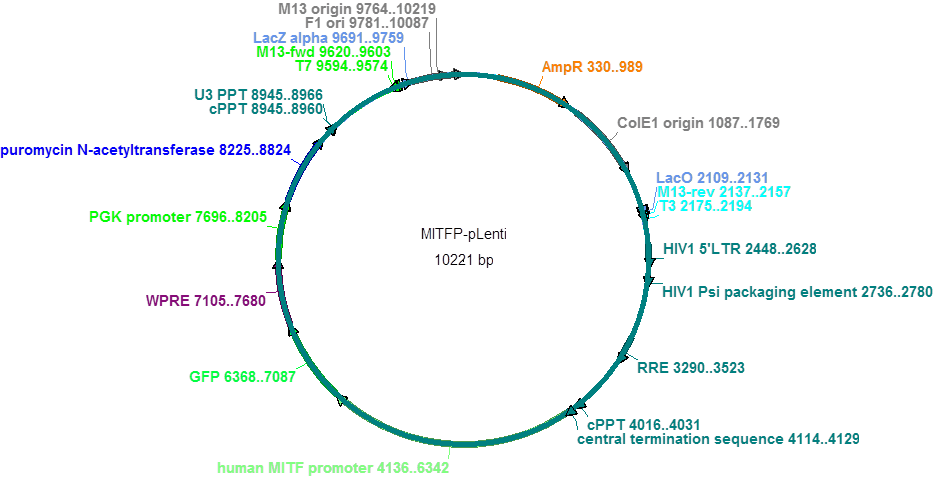
**

**Fig. S2** **Map of the used lentiviral vector MITFP-pLenti.** The GFP gene is located downstream of the MITF promoter. This enables studying the effects of potential transcription factors of MITF by using the fluorescence intensity as the read-out signal. Successfully transfected cells are resistant to the selective marker puromycin due to the puromycin N-acetyltransferase cassette.


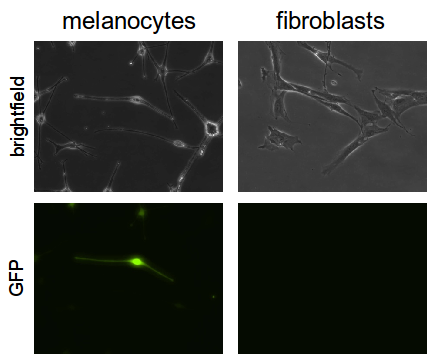
**Fig. S3** **Validation of the functionality of the melanocyte-specific lentiviral reporter vector MITFP-pLenti.** Twelve days after infection with the reporter construct there is reporter activity observable in primary human melanocytes as indicated by a strong GFP signal. No activity of the melanocyte-specific reporter construct was detectable in human fibroblasts 12 days after infection.


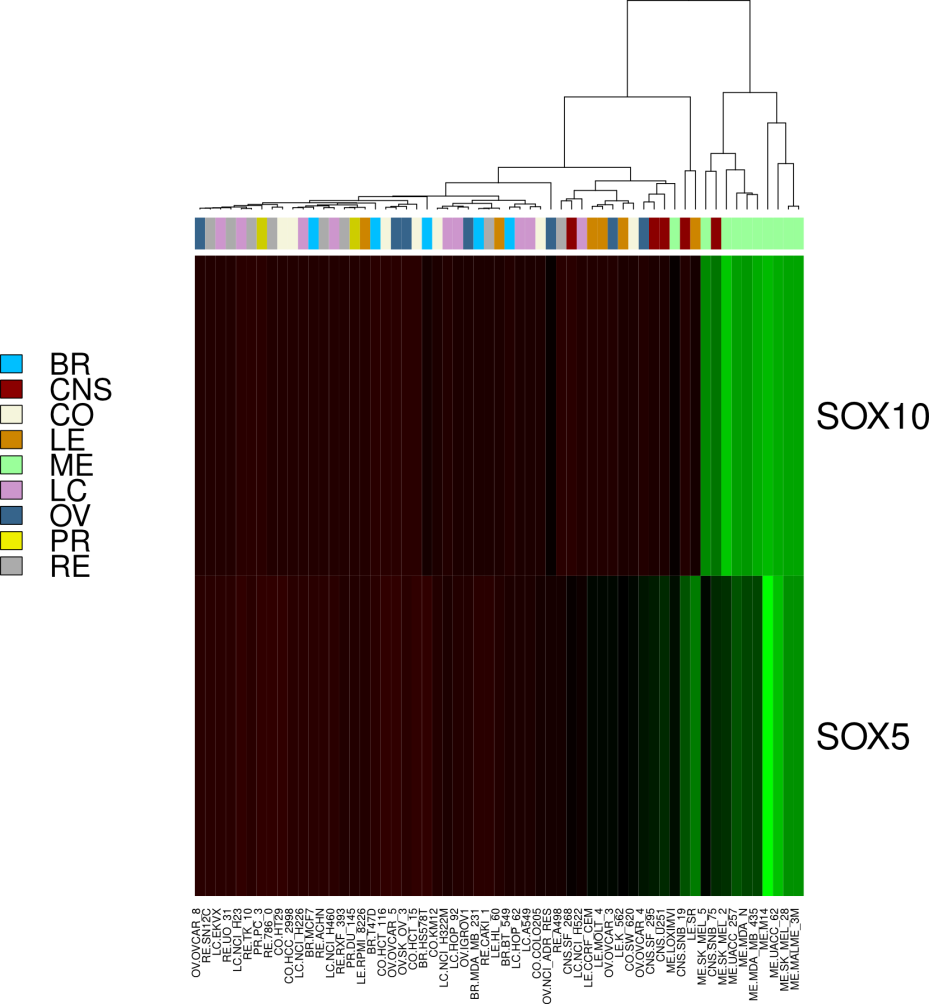

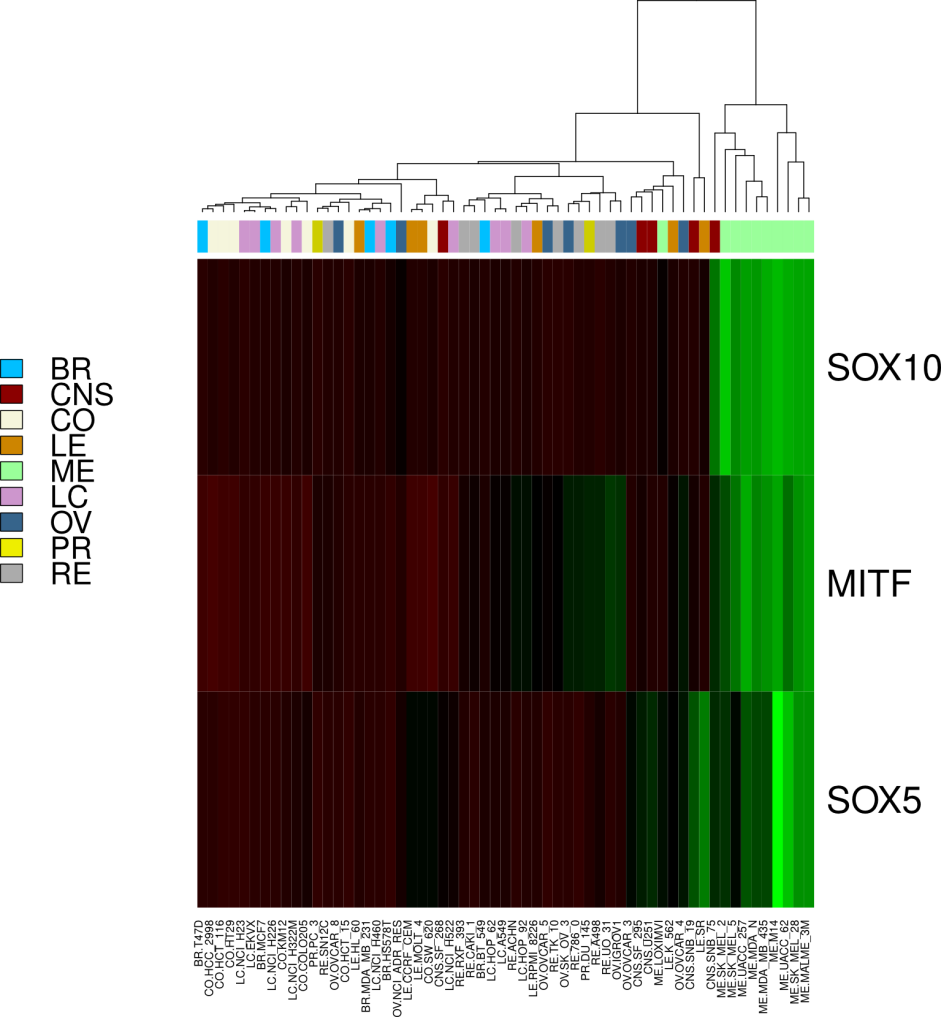


**Fig. S4 Hierarchical cluster analysis of the NCI-60 cell line panel.** The cell lines were clustered based on the expression of SOX5 and SOX10 **(a)** and SOX5, SOX10 and MITF **(b)**. In both cases nine out of ten melanoma samples clustered together.


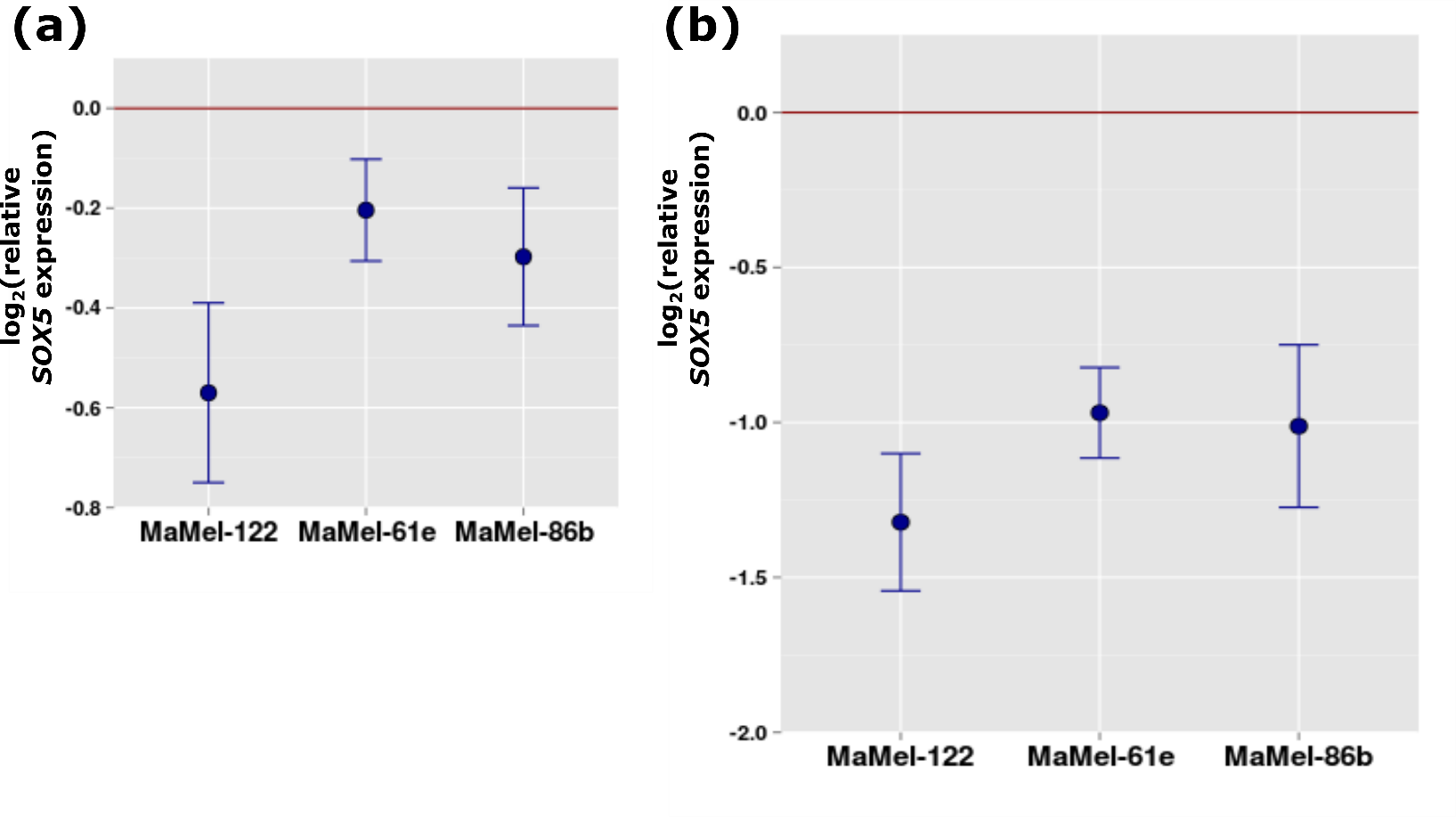


**Fig. S5** **Change in *SOX5* expression 48 h after siRNA transfection.** The melanoma cell lines MaMel-122, MaMel-86b and MaMel-61e were transfected with **(a)** 25 nM *SOX5* siRNA s13303 or **(b)** 10 nM SOX5 siRNA pool. *SOX5* expression was measured by qRT-PCR, normalized to GAPDH expression and control siRNA (a) or control pool siRNA (b) transfected cells. Graphs show the mean expression and standard deviation of fold changes. After transfection knockdown of *SOX5* could be verified for all three cell lines. The knockdown efficiency for the pool was higher compared to the single siRNA.


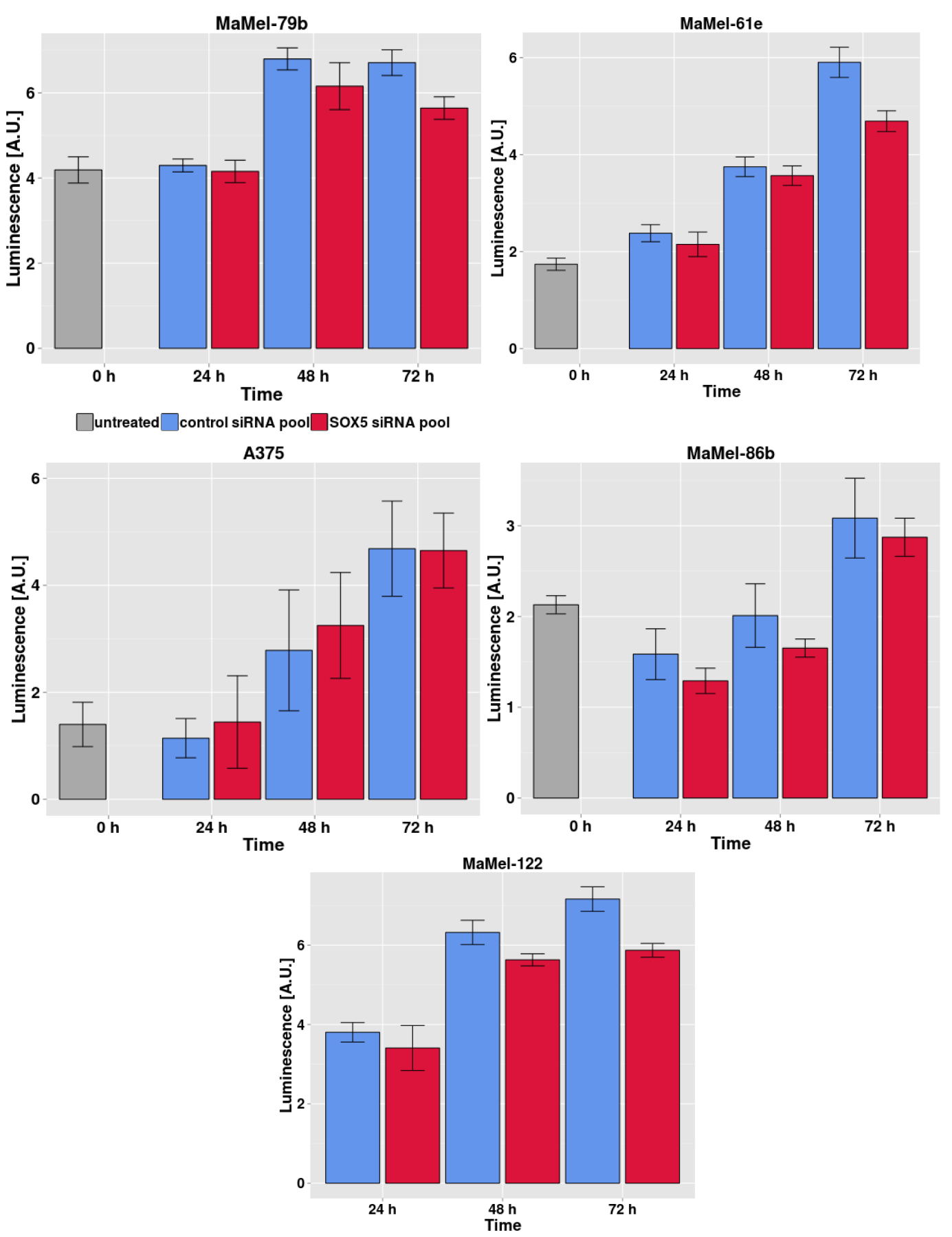


**Fig S6 Cell viability assay after transfection with a pool of siRNA targeting SOX5 and a pool of mock controls.** The cell lines MaMel-79b, MaMel-122, MaMel-61e, MaMel-86b and A375 were transfected with 10 nM SOX5 siRNA pool (red bars) or control (blue bars) siRNA pool. Cell viability was measured 24, 48 and 72 h post transfection. As a reference, cell viability was also measured at the day of transfection (0 h) with untreated cells (grey bars). Mean values of three biological replicates are depicted with SD as error bars.

**Fig S7 Invasion assay after transfection with a pool of siRNA targeting SOX5 and a pool of mock controls.** The cell lines MaMel-79b, MaMel-122, MaMel-61e, MaMel-86b and A375 were transfected with 10 nM SOX5 siRNA pool (red bars) or control (blue bars) siRNA pool. After 48 h cells were transferred to a matrigel coated Boyden chamber plate and invasion was measured after 24 h. Mean values of three technical replicates are depicted with SD as error bars.
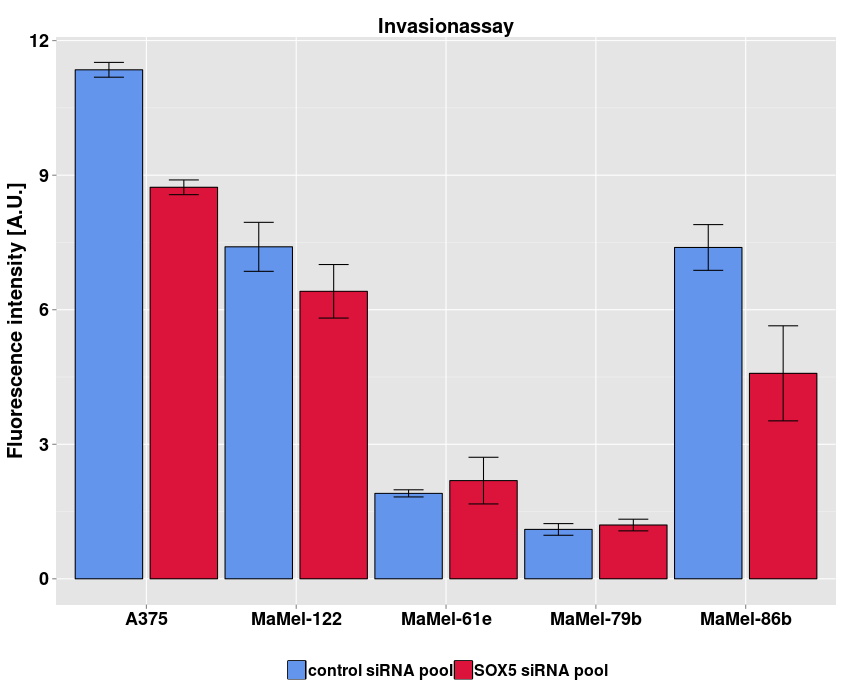


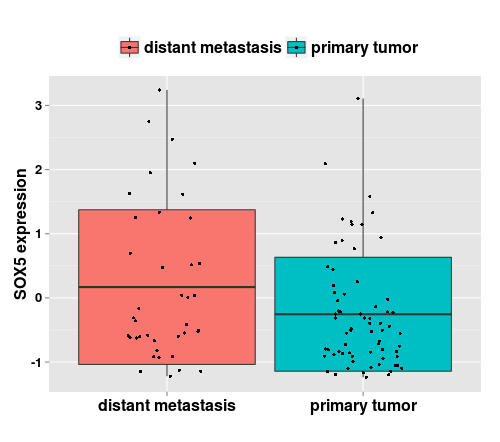
**Fig S8 Boxplot *SOX5* expression for primary and distant metastasis samples.** For the SKCM samples, the expression of *SOX5* was compared for subgroups of primary tumor (69 samples) and distant metastasis (39 samples). The mean *SOX5* expression in primary tumor was -0.25 and showed a lower tendency (p = 0.06; two-sided t-test) compared to distant metastasis with a mean of 0.17.


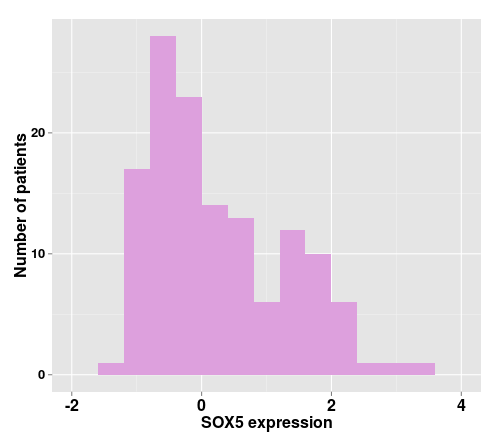


**Fig. S9 Histogram of *SOX5* expression for SCKM samples with vital status dead.**

**
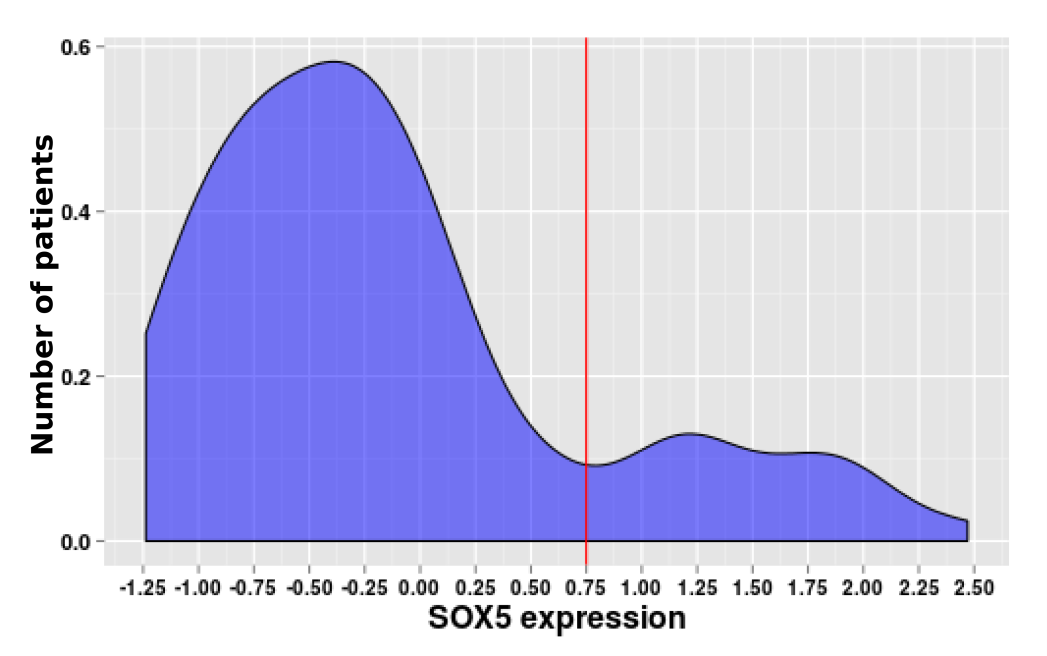
**


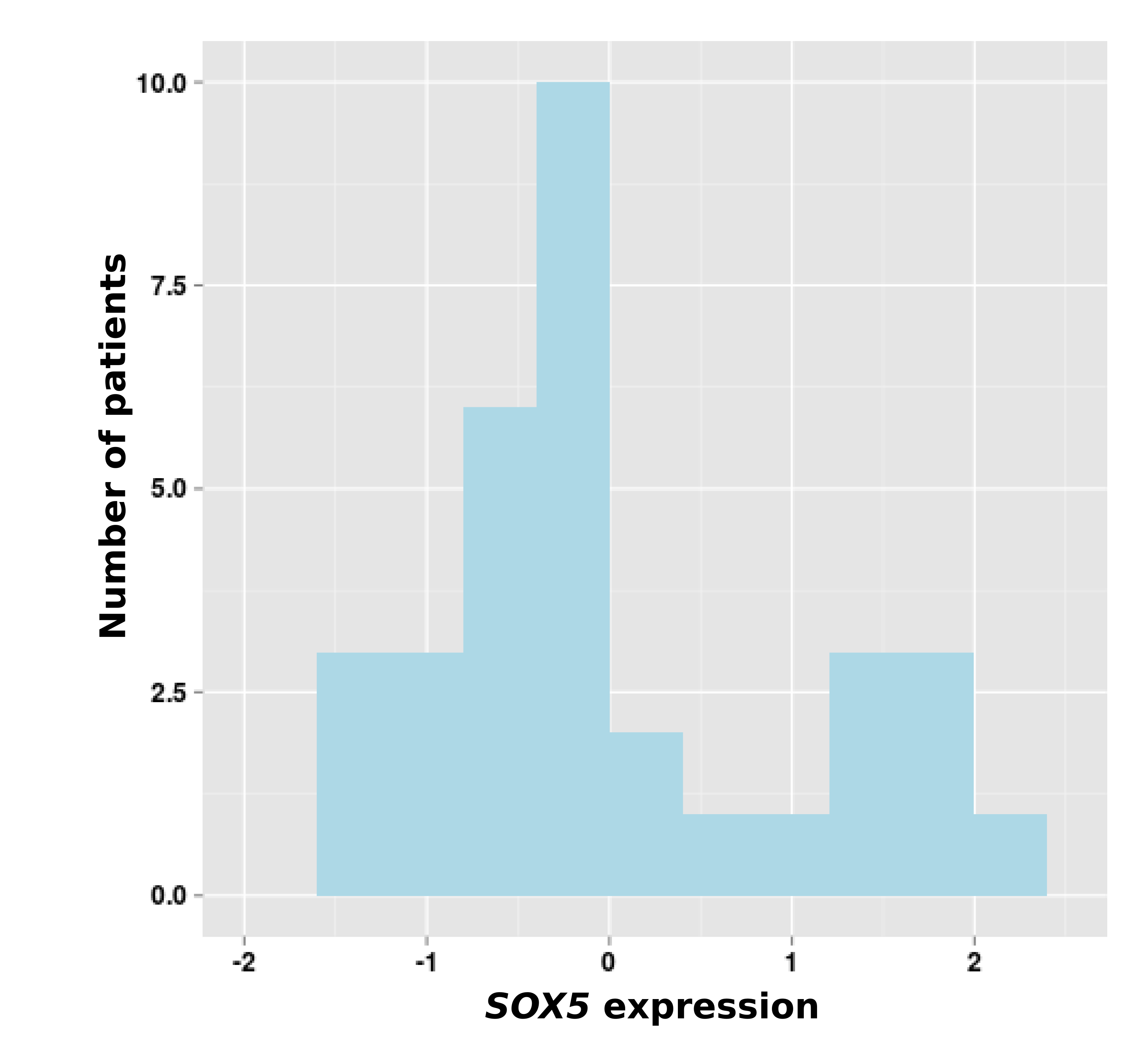

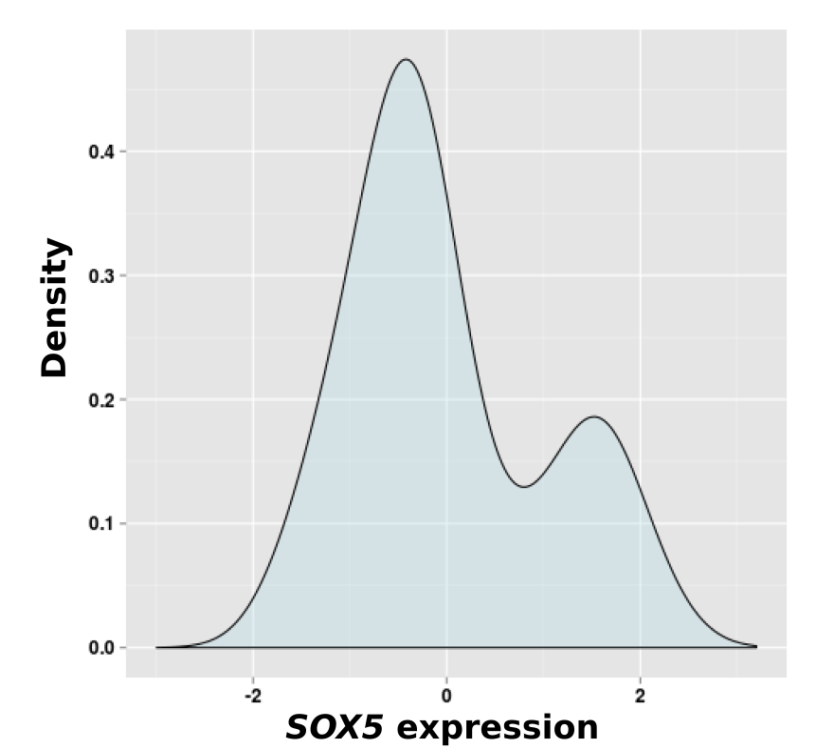
**Fig. S10 Bimodal distribution of *SOX5* expression for thick subgroup of the SKCM samples with Breslow thickness > 4 mm.**

**Fig S11 Density and histogram of *SOX5* expression for melanoma samples from the 33 melanoma cell lines**. *SOX5* expression shows a bimodal distribution.


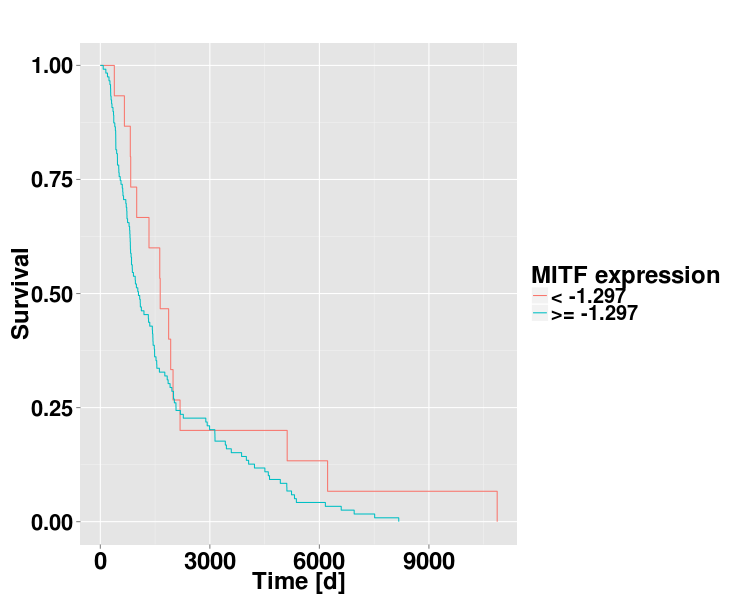


**Fig. S12** **Survival analysis**. The SKCM samples were divided based on their *MITF* expression and, based on their survival times (days to death), a Kaplan-Meier plot was generated. A tendency of better survival for patients with reduced *MITF* was observed, even though it was not significant (P=0.13)


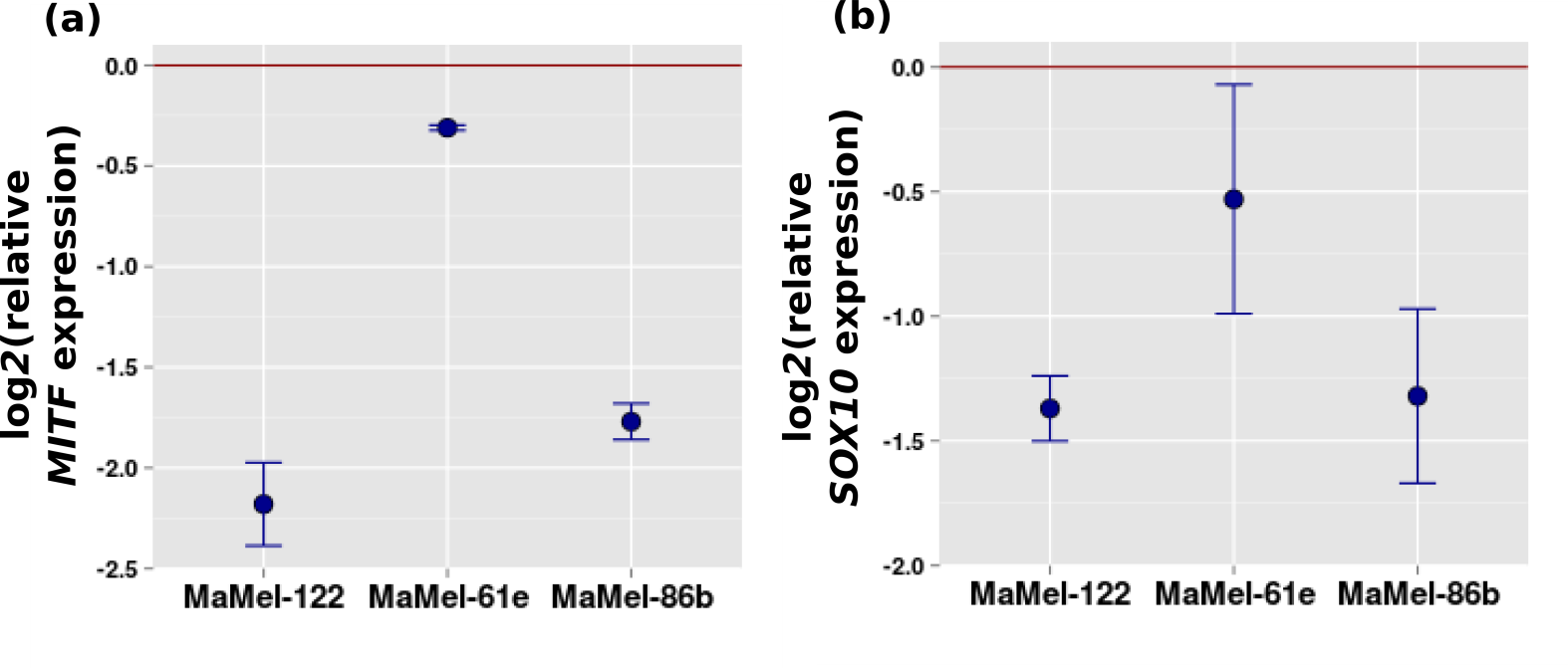
**Fig. S13** **Change in *MITF* (a) and *SOX10* (b) expression 48 h after siRNA transfection.** The melanoma cell lines MaMel-122, MaMel-86b and MaMel-61e were transfected with 25 nM *SOX10* siRNA s13309. *SOX10* and *MITF* expression were measured by qRT-PCR, normalized to GAPDH expression and control siRNA transfected cells. Graphs show the mean expression of four biological replicates and standard deviation of fold changes. After transfection knockdown of *SOX10* could be verified for all three cell lines and the effect on MITF expression could be confirmed for all three cell lines.
